# The *XTH* Gene Family in *Schima superba*: Genome-Wide Identification, Expression Profiles, and Functional Interaction Network Analysis

**DOI:** 10.3389/fpls.2022.911761

**Published:** 2022-06-16

**Authors:** Zhongyi Yang, Rui Zhang, Zhichun Zhou

**Affiliations:** ^1^Research Institute of Subtropical Forestry, Chinese Academy of Forestry, Hangzhou, China; ^2^Zhejiang Provincial Key Laboratory of Tree Breeding, Hangzhou, China

**Keywords:** *XTH* gene family, cell wall remodeling, *Schima superba*, genome-wide identification, expression analysis, functional interaction network

## Abstract

Xyloglucan endotransglucosylase/hydrolase (XTH), belonging to glycoside hydrolase family 16, is one of the key enzymes in plant cell wall remodeling. *Schima superba* is an important timber and fireproof tree species in southern China. However, little is known about *XTHs* in *S. superba*. In the present study, a total of 34 *SsuXTHs* were obtained, which were classified into three subfamilies based on the phylogenetic relationship and unevenly distributed on 18 chromosomes. Furthermore, the intron–exon structure and conserved motif composition of them supported the classification and the members belonging to the same subfamily shared similar gene structures. Segmental and tandem duplication events did not lead to *SsuXTH* gene family expansion, and strong purifying selection pressures during evolution led to similar structure and function of *SsuXTH* gene family. The interaction network and *cis*-acting regulatory elements analysis revealed the *SsuXTH* expression might be regulated by multiple hormones, abiotic stresses and transcription factors. Finally, expression profiles and GO enrichment analysis showed most of the tandem repeat genes were mainly expressed in the phloem and xylem and they mainly participated in glycoside metabolic processes through the transfer and hydrolysis of xyloglucan in the cell wall and then regulated fiber elongation.

## Introduction

Xyloglucan endotransglucosylases/hydrolases (XETs/XEHs, also named XTHs) are xyloglucan-modifying enzymes and belong to glycoside hydrolase family 16. The enzymes have two distinct catalytic activities, XET and XEH, and participate in cell wall relaxation, synthesis and degradation (Campbell and Braam, 1999; Rose et al., 2002; Baumann et al., 2007). Multigene families of *XTHs* have been identified in a variety of plant species, including *Arabidopsis thaliana* (33), *Oryza sativa* (29), *Triticum aestivum* (5), *Sorghum bicolor* (35), *Nicotiana tabacum* (56), *Glycine max* (61), *Solanum lycopersicum* (25), *Actinidia deliciosa* (14), *Malus sieversii* (11), *Gossypium hirsutum* (23), *Brassica rapa* (53), *Brassica oleracea* (38), *B. juncea* (74), and *Populus* spp. (41; Yokoyama and Nishitani, 2001; Yokoyama et al., 2004; Geisler-Lee et al., 2006; Saladié et al., 2006; Liu et al., 2007; Atkinson et al., 2009; Lee et al., 2010; Rai et al., 2016; Nawaz et al., 2017; Song et al., 2018; Wang et al., 2018; Li et al., 2020; Wu et al., 2020; Cheng et al., 2021). Based on their phylogenetic relationships, the *XTH* genes are normally classified into three major groups, Group I, Group II, and Group III, and one minor ancestral group exists differently in species (Yokoyama and Nishitani, 2001; Baumann et al., 2007; Michailidis et al., 2009; Wang et al., 2018; Li et al., 2020; Wu et al., 2020). In some species, groups I and II cannot be separated, such as *OsXTHs* in *O. sativa*, which are clustered into groups I/II and III (Yokoyama et al., 2004). In addition, the members in group III could be subclustered into groups IIIA and IIIB (Baumann et al., 2007). Studies have shown that *XTH* genes in groups I, II and IIIB mainly possess XET activity, whereas group IIIA has XEH activity (Campbell and Braam, 1999; Rose et al., 2002; Baumann et al., 2007).

*XTHs* have spatiotemporal expression specificity and play an important role in plant growth and development. For example, in *A. thaliana*, five genes are expressed in green siliques, two genes are expressed in stems, and at least 10 genes are significantly expressed in the root differentiation zone, promoting cell elongation, expansion, and cell wall formation (Yokoyama and Nishitani, 2001; Maris et al., 2009). Research has revealed that XTH remolds the structure and ductility of the cell wall through bond breaking and glycoside rearrangement in xyloglucan chains (Seale, 2020). At present, several *XTH* genes have been identified to participate in cell wall remodeling in woody plants (Bourquin et al., 2002; Mellerowicz and Sundberg, 2008; Nishikubo et al., 2011; Sundell et al., 2017; Ghosh Dasgupta et al., 2021). For example, *PttXET16A* is involved in the formation of secondary vascular tissue in poplar (*Populus tremula* × *Populus tremuloides*; Bourquin et al., 2002). Overexpression of *PttXET16-34* increases xyloglucan levels in primary-walled xylem and promotes vascular growth and development but decreases in secondary-walled xylem (Nishikubo et al., 2011). XTH activity is positively associated with the Runkel ratio (2 × fiber cell wall thickness/lumen diameter) in *Eucalyptus grandis* (Ghosh Dasgupta et al., 2021). In addition, researchers have found that the higher the XTH activity during the cell elongation stage in cotton, the longer the fiber becomes (Michailidis et al., 2009; Lee et al., 2010; Shao et al., 2011).

*XTH* genes also participate in the response to biotic and abiotic stresses by cell wall remodeling. The *Capsicum annuum* XTH homologous genes *CaXTH1*, *CaXTH2* and *CaXTH3* are upregulated under drought, high salinity and cold temperature, and overexpression of *CaXTH3* enhances tolerance to salt and drought stresses in *A. thaliana* and *Lycopersicon esculentum* (Cho et al., 2006; Choi et al., 2011). *PeXTH* (*Populus euphratica*) could enhance salt tolerance in tobacco plants (Han et al., 2013). *AtXTH31* improves flood resistance in *G. max* (Song et al., 2018). *AtXTH19* also enhances freeze tolerance after cold and subzero acclimation in *A. thaliana* (Takahashi et al., 2021). The promoter activities and expression of *CsXTH1* and *CsXTH3* induced by wounding in *Cucumis sativus* reveal that external mechanical factors play an important role in the regulation of the expression of these genes (Malinowski et al., 2018).

*Schima superba*, belonging to Theaceae, is an evergreen broad-leaved tree species in southern China (Yang et al., 2017; Zhou et al., 2020a). Because of its superior vascular anatomical structures, such as longer fiber and vessel length and thickened secondary cell walls, its timber has great rigidity and toughness and is valued commercially (Li, 2014). *S. superba* grows fast and has a strong ability to resist drought and barrenness and adapt to various environments. Therefore, these characteristics give this species a strong understory regeneration ability with functions in fire prevention. However, little is known about *XTHs* in *S. superba*. In this study, we performed a genome-wide analysis of the *XTH* genes in *S. superba*. From phylogenetic and gene duplication analyses, we revealed the origin and evolution of the *SsuXTH* family; from motif and structural analyses, we demonstrated the conservation of gene structure and function; and from the analysis of *cis*-acting regulatory elements, expression patterns and functional interactions, we predicted the possible functions and regulatory factors of these genes. This is the first time to perform gene family analysis at the genome level of *S. superba*, and it has been found that *XTH* genes may regulate wood fiber elongation. Here, we provide a new insight into the function of *SsuXTH* and lay the foundation for further exploration of the decisive genes of fiber development.

## Materials and Methods

### Genome-Wide Identification of *XTH* Genes

*Schima superba*, its related species *Camellia sinensis*, and the important broad-leaved timber tree species, *Populus* and *E. grandis*, genome sequences were recovered from the *S. superba* genome database (our laboratory, unpublished), Tea Plant Information Archive (TPIA),^1^ *Populus trichocarpa* genome database (version 4.1),^2^ and *E. grandis* genome database (version 2.0).^3^ We downloaded XTH protein sequences of *A. thaliana* from The *Arabidopsis* Information Resource (TAIR)^4^ and used them as the query sequences to scan the *Populus*, *E. grandis*, *C. sinensis*, and *S. superba* genome sequences using the Protein Basic Logical Alignment Search Tool (BLASTP, United States National Library of Medicine) with an e-value (≤1e^−5^) and an identity match (≥50%) as thresholds (Cui et al., 2019). To further confirm the presence of domains, we also conducted a hidden Markov model (HMM) search for sequence homologs using the HMMER 3.0 program (Mistry et al., 2021),^5^ and XET_C (PF06955.12) and Glyco_hydro_16 (PF00722.21) were used as baits. The BLASTP and HMM search results were then integrated to identify candidate *XTH* genes. Their sequences were then submitted to the NCBI conserved domains database (CDD; Lu et al., 2020)^6^ to verify the presence of the XET and GH16 domains (Zhu et al., 2020).

### Sequence Analysis of XTH Proteins

The physicochemical properties of proteins encoded by XTH proteins in *Populus*, *E. grandis, C. sinensis and S. superba*, including molecular weight (MW), isoelectric point (PI), instability index, grand average of hydropathy (GRAVY) values and so on, were calculated using the ExPASy online tool (Wilkins et al., 1999).^7^ Finally, the subcellular localization of XTH proteins was predicted using Euk-mPLoc 2.0 (Chou and Shen, 2010).^8^

### Phylogenetic Analysis of XTH Proteins

The XTH peptide sequences from *P. trichocarpa* (Ptri), *E. grandis* (Egr), *C. sinensis* (Cs), *S. superba* (Ssu) and *A. thaliana* (At) were aligned by using ClustalW (Larkin et al., 2007) with default parameters. The aligned sequences were then used to generate the phylogenetic tree using MEGA7.0 software (Kumar et al., 2016). The tree was constructed using the neighbor-joining (NJ) algorithm with default parameters. The reliability of the phylogenetic tree was analyzed by the bootstrap method, and replicates were set to 1,000. The resulting tree was visualized using EvolView v3 (Subramanian et al., 2019).^9^

### Conserved Motif and Gene Structure Analyses

In order to identify the conserved motifs, the MEME (Bailey et al., 2015)^10^ suite was used with default parameters. Exons and introns of each *XTH* were identified using the gff3 file of the *S. superba* genome, which contained information regarding gene structure, and visualized using TBtools (Chen et al., 2020).

### Chromosomal Localization and Ka/Ks Calculation

The chromosomal location information of the *XTH* gene family and the length of each chromosome were extracted using the annotated files of the *S. superba* genome. The gene positions on chromosomes were drafted by using TBtools (Chen et al., 2020).

And paralogous genes were determined by aligning and phylogenetically analyzing XTH proteins (He et al., 2016; Zhou et al., 2020b). Genes with a distribution on the chromosome in the range of 100 kb and separated by less than five genes were considered to be tandem duplicates according to the same standard as in rice (TIGR Rice Genome Annotation).^11^

Furthermore, the Ka/Ks ratios were evaluated using TBtools software (Chen et al., 2020) to assess the synonymous and nonsynonymous groups. The Ks values represent the divergence time of duplication events, and the Ka/Ks values represent the selective pressure of duplicate genes. Divergence time (T) was calculated by Ks/2r × 10^−6^ million years ago (Mya; Khan et al., 2018), where the *r* is the rate of divergence. For Theaceae plants, *r* = 5.62 × 10^−9^(Xia et al., 2020). In general, Ka/Ks < 1.0 represents purifying or negative selection, Ka/Ks = 1.0 represents neutral selection, and Ka/Ks > 1.0 represents positive selection (Zhang et al., 2006).

### *cis*-Regulatory Element Analyses

The promoter sequences of 2,000 bp of the *XTH* genes were retrieved from the *S. superba* genome database (unpublished) to analyze the *cis*-acting regulatory elements (CAREs). PlantCARE 10 (Lescot et al., 2002)^12^ was used for identifying and analyzing the CAREs.

### Functional Interaction Analyses

The protein interaction network was generated from the STRING database (Szklarczyk et al., 2019)^13^ based on an *Arabidopsis* association model with default parameters. The *Arabidopsis* model had to be employed due to the absence of the *S. superba* database in the STRING server. And the homologous proteins of *Arabidopsis* were designated STRING proteins and were selected based on high bit scores in the BLAST results (Chatterjee et al., 2020).

### Structural Prediction of XTH Proteins

TMHMM Sever v2.0 (Krogh et al., 2001)^14^ was used to predict transmembrane helices (TMHs), SignalP-5.0 (Armenteros et al., 2019)^15^ was used to predict signal peptides, and NetPhos-3.1 (Blom et al., 1999)^16^ was used to predict phosphorylation sites in the amino acid sequence of each SsuXTH protein. We used the online software SOPMA (Levin et al., 1986; Levin and Garnier, 1988)^17^ and SWISS-MODEL (Waterhouse et al., 2018)^18^ to predict the secondary and three-dimensional (3D) structures of XTH proteins.

### Gene Expression Profile Analysis and Quantitative Real-Time PCR

In August 2020, different tissues were collected for tissue-specific expression profile analysis from the germplasm bank of *S. superba* clones (119°06′E, 28°03’N) in Zhejiang Longquan. The tissues, including xylem, phloem, mature leaves, buds, fruits, and roots of tissue culture seedlings sub-cultured for 60 days (Yang et al., 2021). Each sample was set up in biological triplicates, and all samples were frozen with liquid nitrogen and stored at −80°C.

Total RNA was extracted from the different tissues of *S. superba* using the Easy Plant RNA Kit (Polysaccharides & Polyphenolics-rich; Code No. DR0407050, Easy-Do, China). Then, RNA samples with A260/A280 ratios between 1.8 and 2.1 were used to synthesize first-strand cDNA with PrimeScriptTM RT master mix (Perfect Real Time; Code No. RR036A, Takara, Japan). Quantitative real-time PCR (qRT–PCR) was performed using 2X TB Green Premix Ex Taq II (Tli RNase H Plus; Code No. RR820A, TaKaRa, Japan) and Applied Biosystems Q7 (United States). We used a previously described reaction system and qRT–PCR procedure, and *SsuACT* (*Ssu18G01981*) was used as an internal reference gene to normalize the gene expression level (Yang et al., 2021). The primers are listed in Supplementary Table 1.

### Transcriptome Data and GO Classification Analysis

For the identification of cell wall remodeling- and fiber development-related *XTH* genes, we used RNA-seq data from three materials, which were secondary xylem, phloem and cambium of three 12-year-old half-sib progenies (S1, S2 and S3) from SC25 family in *S. superba* with different vascular characteristics. Specifically, the fiber length of S2 was significantly greater than that of S1 and S3, and there was no significant difference between those of S1 and S3 (*p* > 0.01). The cell wall thickness of S2 was significantly greater than that of S3 and was not significantly different from that of S1 (*p* > 0.01). The RNA-seq data in this study were deposited in the NCBI Sequence Read Archive (SRA) database under numbers SRR18212748 ~ SRR18212756.

Fragments per kilobase of transcript per million mapped reads (FPKM) values of *SsuXTHs* were used to evaluate transcript abundance. DESeq2 (Love et al., 2014) was used to conduct differential gene expression analysis, and gene ontology (GO) classification analysis was performed based on the differential gene expression analysis by the clusterProfiler R package (Wu et al., 2021). Finally, heatmaps of *SsuXTH* expression patterns were drawn by TBtools software (Chen et al., 2020).

### Statistical Analyses

The average Ct value was calculated from three biological replicates and three technical replicates. Relative gene expression levels were calculated using the 2^−△△Ct^ method (Livak and Schmittgen, 2001). Significance differences were determined by one-way analysis of variance with SPSS (Statistical Package for the Social Sciences) version 17.0 (Chicago, IL, United States).

## Results

### Identification and Sequence Analysis of *XTH* Genes

In our study, *XTH* genes were identified from the *S. superba*, *C. sinensis*, *P. trichocarpa* and *E. grandis* genomes. Compared with *A. thaliana* (33), *P. trichocarpa* (38)*, E. grandis* (38) and *C. sinensis* (34), the *XTH* family in *S. superba* (34) was not obviously contracted or expanded (Supplementary Tables 2, 3).

The sequences of XTH proteins in the five plant species were similar (Table 1; Supplementary Table 4). Acidic proteins (PI < 7) accounted for 47.46%, alkaline proteins (PI > 7) accounted for 51.98%, and the remaining 0.56% were neutral proteins (PI = 7; AT4G37800.1_Atha). Stable (<40) and unstable (>40) proteins accounted for 47.46 and 52.54%, respectively. Most were hydrophilic in nature (99.44%), and only one gene corresponded to a hydrophobic protein (TEA007187.1_Csin). The aliphatic index of the peptides ranged from 43.28 (SsuXTH13) to 101.33 (TEA007187.1_Csin).

**Table 1:** Statistics of the physicochemical properties of the *XTH* gene family in five plant species.

Species	Polypeptide length (aa)	Molecular weight (MW, kDa)	PI	Instability index	Aliphatic index	GRAVY	Subcellular localization			
							Cell wall	Cell wall and Cytoplasm	Nucleus	Extracell
*S. superba*	138–367	16.11–41.31	4.71–9.36	32.27–58.52	43.28–80.06	<0: 34	18	12	0	4
			<7: 14	<40: 13						
			>7: 20	>40: 21						
*A. thaliana*	269–357	30.76–41.21	5.05–9.53	23.24–56.4	55.64–76.9	<0: 33	23	10	0	0
			<7: 9	<40: 23						
			>7: 23	>40: 10						
			=7: 1							
*C. sinensis*	178–616	20.27–68.94	4.76–9.64	23.33–52.88	56.97–101.33	<0: 33 >0: 1	13	15	1	5
			<7: 19	<40: 14						
			>7: 15	>40: 20						
*P. trichocarpa*	214–367	24.83–41.63	4.47–9.67	27.44–55.83	54.66–74.93	<0: 38	20	18	0	0
			<7: 17	<40: 20						
			>7: 21	>40: 18						
*E. grandis*	261–356	30.05–40.80	4.7–9.56	25.06–56.26	58.16–76.86	<0: 38	15	18	0	5
			<7: 25	<40: 14						
			>7: 13	>40: 24						

In *S. superba*, the polypeptide lengths and molecular weights of SsuXTH were short and low, especially those of SsuXTH22, compared with those of the other four plants. The number of alkaline proteins was greater than that of acidic proteins, consistent with results in *A. thaliana* and *P. trichocarpa*. The number of stable proteins was less than that of unstable proteins, consistent with results in *C. sinensis* and *E. grandis*.

Furthermore, the subcellular localization results of the XTH proteins in the five plant species were similar, and most were localized in the cell wall (Table 1; Supplementary Table 4). All XTH proteins of *A. thaliana* and *P. trichocarpa* were localized in the cell wall. However, 30, 28 and 33 XTH proteins were localized in the cell walls of *S. superba, C. sinensis* and *E. grandis*, respectively. This indicated that most XTH proteins in the five plants played an important role in cell wall remodeling.

### Phylogenetic Analysis of XTH Proteins

**Figure 1:**
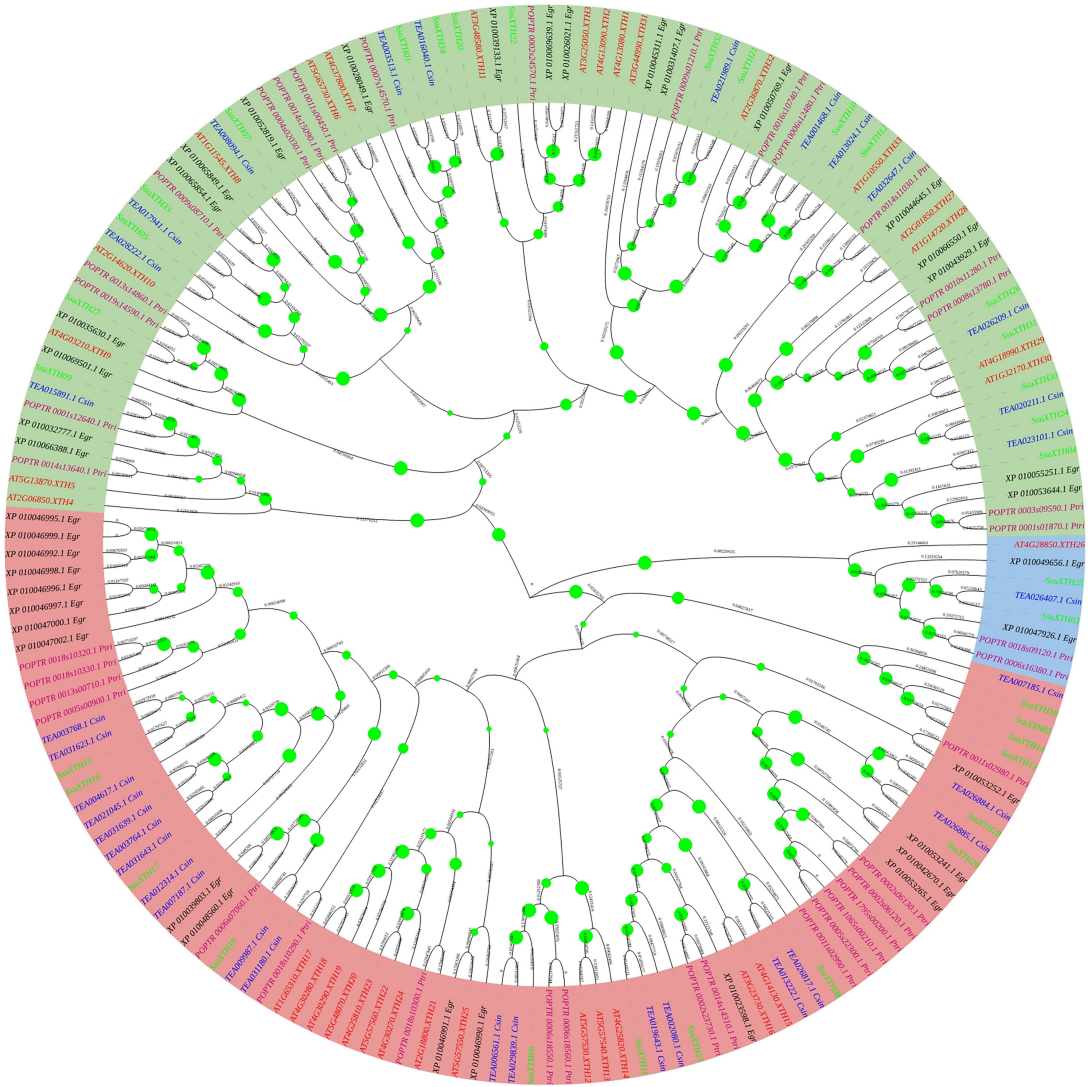
Phylogenetic tree of XTH proteins based on the full-length protein sequences using the neighbor-joining method. The three different groups are indicated by different background colors. The proteins of *S. superba,* tea plant, poplar, *A. thaliana* and *E. grandis* are indicated by different font colors.

According to the topological structure and sequence homology of the phylogenetic tree, the 177 XTH proteins were divided into three evolutionary branches, named Clade I (green), Clade II (pink) and Clade III (blue; Figure 1). From the tree, we found that Clade I had the largest number of proteins (86 out of 177), and the correlation between protein clustering and species was weak, indicating that the XTH proteins in the branch was primitive and less conserved, or the gene origin occurred before the differentiation of the five plants; the XTH proteins in Clade II (83 out of 177) were mostly clustered by species, indicating that the XTH proteins in this branch evolved late and were highly conserved; Clade III had the fewest members of the XTH proteins family, with only 8 proteins. In addition, it could be seen that two woody plants of Theaceae, *S. superba* and *C. sinensis,* often gathered in the same branch, while poplar and *E. grandis* were relatively closely related. It should be noted that the ancestral group included AtXTH1, AtXTH2, AtXTH3, AtXTH11, POPTR_0002s24570.1_Ptri, XP_010026021.1_Egr, XP_010039133.1_Egr, XP_010069639.1_Egr, SsuXTH20 and SsuXTH22, according to previous research (Baumann et al., 2007), and it was located in Clade I.

**Figure 2:**
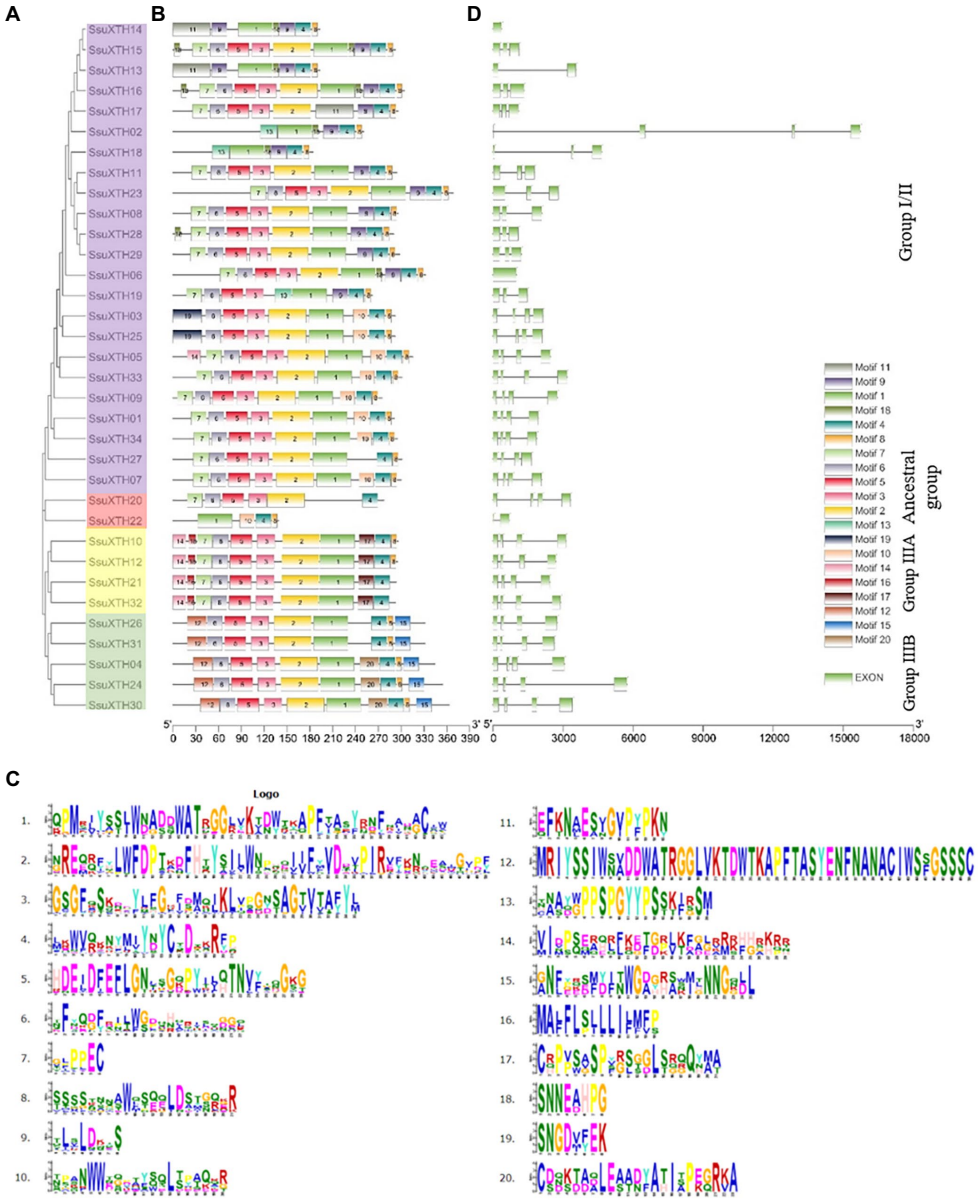
Evolutionary analysis, motif compositions, and gene structures of 34 SsuXTHs. **(A)** The phylogenetic tree of SsuXTHs, and four different groups are indicated by different background colors. **(B)** The motif location in SsuXTHs. **(C)** The gene structures of SsuXTHs. **(D)** The legend depicting the protein sequence of the corresponding motif.

Then, we divided the SsuXTHs into three groups according to the clustering branches of the phylogenetic tree (Ancestral group, Group I/II and Group III; Figure 2A), and group III was also divided into two branches (Group IIIA and Group IIIB). Then, the branches showed a direct relationship with the subcellular localization. For example, the ancestral group had only 2 proteins (SsuXTH20 and SsuXTH22) localized in extracellular spaces; Group III had 9 proteins localized in the cell wall; and Group I/II had 23 proteins localized in the cell wall (9 out of 34), extracellular space (2 out of 34) and cell wall and cytoplasm (12 out of 34).

### Conserved Motif and Gene Structural Analyses

We detected 20 conserved motifs among SsuXTHs, and each motif appeared in 2–34 proteins (Figures 2B,C). Notably, motif 5 widely existed in Groups I/II and III, and DEIDFEFLG was the conserved motif that catalyzed the enzymatic reaction of XET and the characteristic motif of this family. Motif 4 existed in all SsuXTH proteins, indicating that this core conserved motif was also very important for XTH proteins, which meant that SsuXTH proteins had similar or the same structure and function. In addition, we also found that motifs 12, 14, 15, 16, 17 and 20 existed only in Group III, indicating that these motifs might have evolutionary specificity in the members of their group. In general, the motifs were highly conserved, which indicated that the functions of SsuXTH proteins were relatively similar.

To identify the structural characteristics of the *SsuXTHs*, the intron/exon architecture of the genes was analyzed using TBtools (Figure 2D). Analyzing these intron arrangements provides significant information regarding the evolution, regulation, and function of the *XTHs* (Liu et al., 2015). All the genes of Group III contained three introns and four exons. However, the numbers of introns and exons in the ancestral group and Group I/II were inconsistent.

### Chromosomal Localization and Ka/Ks Calculation

**Figure 3:**
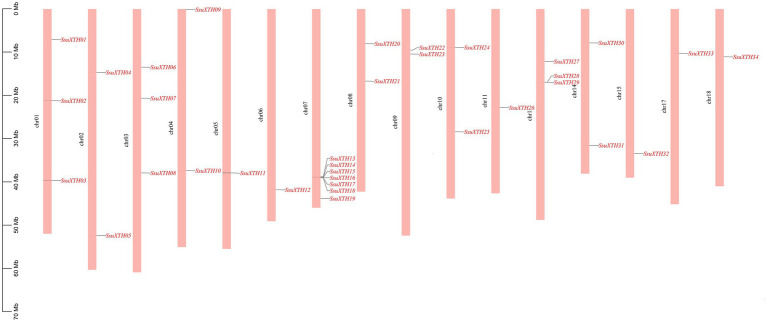
The chromosomal distribution of *SsuXTH* genes. The left scale represents the length of the chromosomes.

The 34 *SsuXTHs* were unevenly mapped in the 18 chromosomes of *S. superba* (Figure 3). Chromosomes 12 and 16 did not carry any *XTH* genes. Among the rest, chromosome 7 contained the largest number (7; 20.59%) of *SsuXTH* genes, followed by chromosomes 1, 3 and 13, which contained three members (8.82%). Chromosomes 2, 4, 8, 9, 10 and 14 contained two members (5.88%), and the other chromosomes only contained one member. In addition, there was no evidence suggesting a positive correlation between the gene number and chromosome length.

Moreover, we found two gene clusters (Figure 3). One was located on chromosome 7, including five *XTH* genes (*SsuXTH14-18*). The other was on chromosome 13, including two *XTH* genes (*SsuXTH28-29*). Thus, tandem repeats may be one of the important mechanisms of *SsuXTH* gene expansion. Gene duplication has long been considered one of the main forces in the evolution and expansion of the gene family (Crow and Wagner, 2006). Therefore, we investigated the different types of gene duplications in the *SsuXTH* gene family. According to phylogenetic analysis, we found 11 *XTH* paralogous gene pairs (Figure 2), with a proportion of 64.5%. We inferred that segmental gene duplication may be another important mechanism of *SsuXTH* gene expansion. Additionally, analyzing the amino acid sequence identity of paralogous gene pairs found that 10 paralogous pairs of *SsuXTHs* displayed high amino acid sequence identities (63.7–93.3%). On the flip side, the sequence identity of *SsuXTH*20/*SsuXTH*22 displayed lower levels (16.7%; Table 2); of course, their gene structures and conserved motifs were significantly different.

**Table 2:** Sequence alignments of the 11 paralogous gene pairs of *XTH* genes in *S. superba*.

Paralogous pairs	Score	Identities	Positives	Gaps
*SsuXTH14/SsuXTH15*	1,493	280/300 (93.3%)	286/300 (95.3%)	8/300 (2.7%)
*SsuXTH11/SsuXTH23*	1305.5	244/372 (65.6%)	265/372 (71.2%)	82/372 (22.0%)
*SsuXTH28/SsuXTH29*	1,045	193/303 (63.7%)	234/303 (77.2%)	18/303 (5.9%)
*SsuXTH03/SsuXTH25*	1,396	251/292 (86.0%)	270/292 (92.5%)	0/292 (0.0%)
*SsuXTH05/SsuXTH33*	1,235	226/315 (71.7%)	257/315 (81.6%)	14/315 (4.4%)
*SsuXTH01/SsuXTH34*	1,405	252/296 (85.1%)	273/296 (92.2%)	6/296 (2.0%)
*SsuXTH20/SsuXTH22*	204	49/293 (16.7%)	77/293 (26.3%)	170/293 (58.0%)
*SsuXTH10/SsuXTH12*	1,484	261/296 (88.2%)	282/296 (95.3%)	1/296 (0.3%)
*SsuXTH21/SsuXTH32*	1,380	245/293 (83.6%)	265/293 (90.4%)	1/293 (0.3%)
*SsuXTH26/SsuXTH31*	1,493	279/332 (84.0%)	302/332 (91.0%)	2/332 (0.6%)
*SsuXTH24/SsuXTH30*	1,516	293/366 (80.1%)	320/366 (87.4%)	15/366 (4.1%)

Additionally, the Ka/Ks ratios were calculated to understand the evolutionary pressure and gene divergence mechanism. The Ka/Ks ratio helps determine whether Darwinian selection pressures are involved in duplication events (Tian et al., 2017; Chatterjee et al., 2020). If the value of the Ka/Ks ratio is >1, it implies positive or Darwinian selection, and the gene is more prone to nonsynonymous mutation. If the ratio is equal to 1, it implies neutral selection, and if the ratio is <1, it determines purifying selection, and the gene is more prone to synonymous mutation (Bowers et al., 2003; Liu et al., 2014). The results showed that the Ka/Ks ratios of all gene pairs were less than 1 (Table 3), indicating negative or purifying selection.

**Table 3:** Estimated duplication times of *XTH* paralogous gene pairs in *S. superba*.

Paralogous pairs	Ka	Ks	Ka/Ks	Date (Mya)
*SsuXTH14/SsuXTH15*	0.089966	0.308826	0.291315	27.48
*SsuXTH11/SsuXTH23*	0.089845	0.607487	0.147896	54.05
*SsuXTH28/SsuXTH29*	0.224866	1.367578	0.164427	121.67
*SsuXTH03/SsuXTH25*	0.078484	0.315650	0.248644	28.08
*SsuXTH05/SsuXTH33*	0.155537	0.714642	0.217643	63.58
*SsuXTH01/SsuXTH34*	0.067106	0.728438	0.092123	64.81
*SsuXTH20/SsuXTH22*	0.604337	3.617050	0.167080	321.80
*SsuXTH10/SsuXTH12*	0.061322	0.457340	0.134083	40.69
*SsuXTH21/SsuXTH32*	0.091961	0.609521	0.150875	54.23
*SsuXTH26/SsuXTH31*	0.079176	0.401399	0.197250	35.71
*SsuXTH24/SsuXTH30*	0.106329	0.401642	0.264736	35.73

Finally, we estimated the replication time of *S. superba XTH* paralogous gene pairs according to the Ks value (Table 3), in which the replication time of *SsuXTH20*/*SsuXTH22* was 321.80 Mya, which was close to the origin time of gymnosperms, and the resulting times were very ancient. Interestingly, the gene pairs were also located in the ancestral group by phylogenetic analysis. The replication time of the *SsuXTH28*/*SsuXTH29* gene pair was 121.67 Mya, which was in the Early Cretaceous. It was reported that the *Schima* genus possibly first appeared by the Late Cretaceous and continued to differentiate and expand during the Tertiary and probably first appeared in Asia (its modern distribution area) by the Late Oligocene (Shi et al., 2017). Therefore, the replication time of most gene pairs occurred between 27.48 and 64.81 Mya.

### Analysis of *cis*-Acting Regulatory Elements

**Figure 4:**
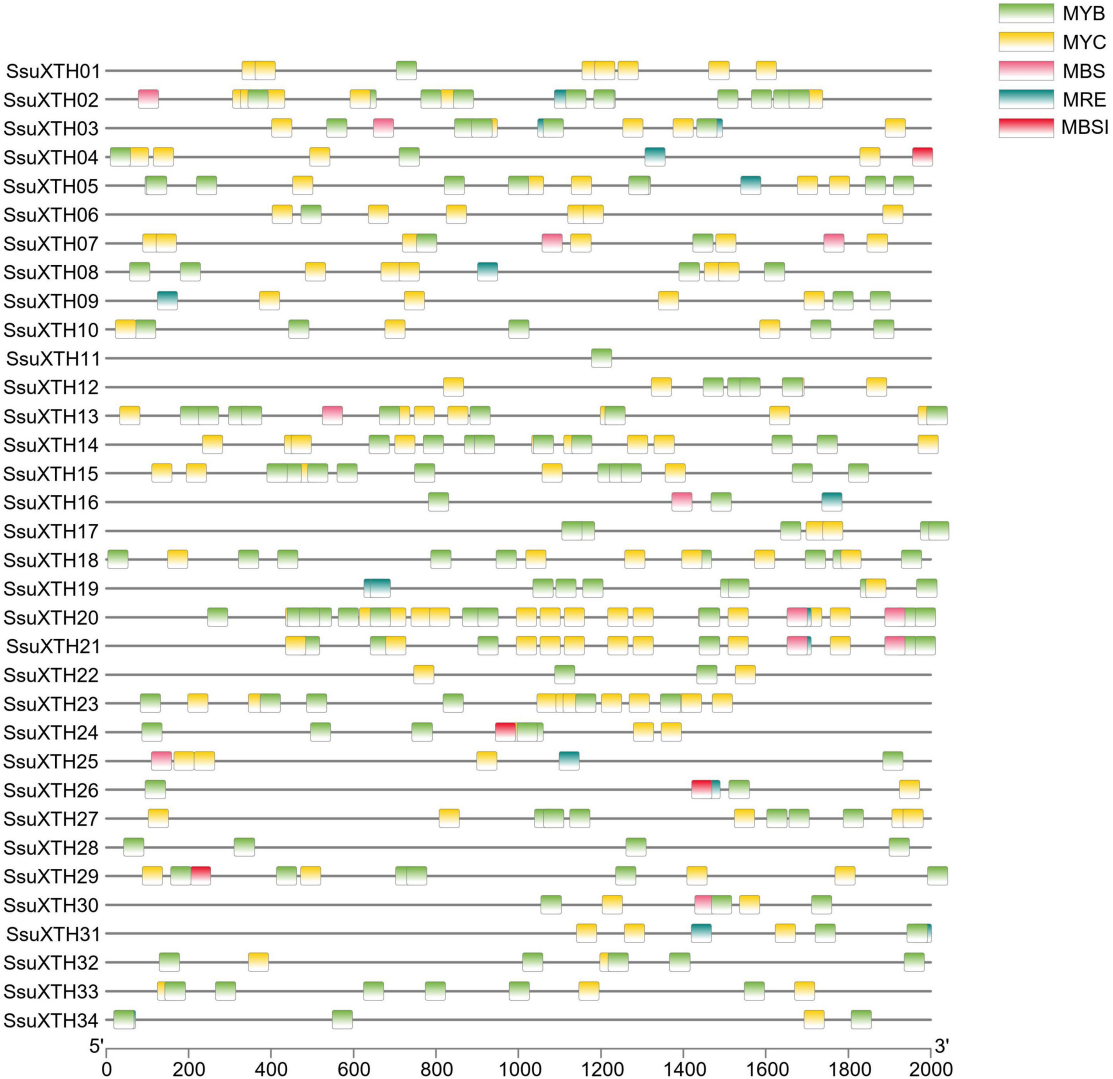
Analysis of the MYB and MYC binding sites in the promoter regions of *SsuXTH* genes. The 2-kb sequences of *SsuXTH* gene promoter regions were extracted and analyzed, and the different binding sites were color-coded. MBS: MYB binding site involved in the drought response; MRE: MYB binding site involved in light responsiveness; MBSI: MYB binding site involved in flavonoid biosynthetic gene regulation.

Various *cis*-acting regulatory elements were detected in the promoter regions of *SsuXTH* genes (Supplementary Tables 5, 6; Figure 4). Phytohormone responsive elements, such as ABREs (abscisic acid responsive elements), AuxREs (auxin responsive elements), GAREs (gibberellin responsive elements), MeJAREs (MeJA responsive elements) and SAREs (salicylic acid responsive elements), were included in the promoter regions, suggesting that the expression of *SsuXTH* genes might be regulated by multiple phytohormones. Additionally, some stress-related *cis*-acting elements, such as DSREs (drought and stress responsive elements) and LTREs (low-temperature responsive elements), were also found in the *SsuXTH* promoter regions, and these results indicated that *SsuXTH* genes might be closely related to the responses to multiple abiotic stresses. Moreover, each *SsuXTH* contained multiple copies of LREs (light responsive elements). Notably, the promoter region of *SsuXTH* genes contained some transcription factor-binding sites, such as those of MYBs, MYCs, HD-Zips, and GATAs. In particular, multiple MYB binding sites appeared in all *SsuXTHs* (Figure 4).

### Structural Prediction of SsuXTH Proteins

To study the functions of SsuXTH proteins, we predicted their transmembrane helices (TMHs), signal peptides and phosphorylation sites (Supplementary Table 7). Seventeen SsuXTH proteins predicted by TMHMM had only one TMH, and the TMHs might be a signal peptide. Signalp-5.0 predicted that 24 SsuXTH proteins had signal peptides, and the probability of signal peptides in 16 genes was greater than 0.9. The results predicted by the two tools were not completely consistent, possible because of the different algorithms that they employed.

Netphos-3.1 prediction results (Supplementary Table 7) showed that the number of phosphorylation sites of 34 SsuXTH proteins varied from 18 to 81, among which the number of SsuXTH22 was the lowest, and that of SsuXTH30 was the highest. In addition, except for SsuXTH09, SsuXTH22 and SsuXTH34, the numbers of serine residue sites were significantly greater than those of the other two amino acid residues of the other proteins.

The secondary structures of SsuXTH proteins were rich, including alpha helix, beta turn, extended strand and random coil structures (Supplementary Table 8). Among them, the random coil was the most common (46.39% ~ 57.29%), and the beta turn was the least common (3.93% ~ 7.58%). The quantitative order was random coil > extended strand/alpha helix > beta turn for the secondary structures in all proteins, indicating that the structures and functions of the SsuXTH proteins were conserved.

3D proteins could be effectively used for understanding the structure and mode of action of XTH enzymes, which will help reveal the functions of SsuXTHs. The structural properties of all SsuXTHs were displayed in homology-based tertiary (3D) protein models (Supplementary Table 9), which were predicted *via* the SWISS-MODEL website. Predicted models were based on the reported templates to heuristically maximize the alignment coverage, percentage identity, and confidence score for the tested sequences. All 3D protein models were constructed with 38–91% seq-identity, and the residue coverage varied from 63 to 99%, suggesting that structure prediction of SsuXTH proteins was highly reliable. Most SsuXTH proteins contained similar structures, especially those from the same branch, implying that SsuXTH proteins may evolved from same ancestor sequence and/or under purification selection force to keep stabilization during long-term acclimation after the initially divergent (Zhu et al., 2019). For example, the 3D structure of proteins from Group IIIA were same. In addition, the 3D structure of SsuXTH13, SsuXTH18 and SsuXTH22 were quite different from that of other proteins, and they were in different groups, but their polypeptide lengths and molecular weights were shortest and lowest. These predicted 3D models provided an important basis for the functional analysis of the XTH proteins.

### Tissue-Specific Expression Profiles of *SsuXTHs*

**Figure 5:**
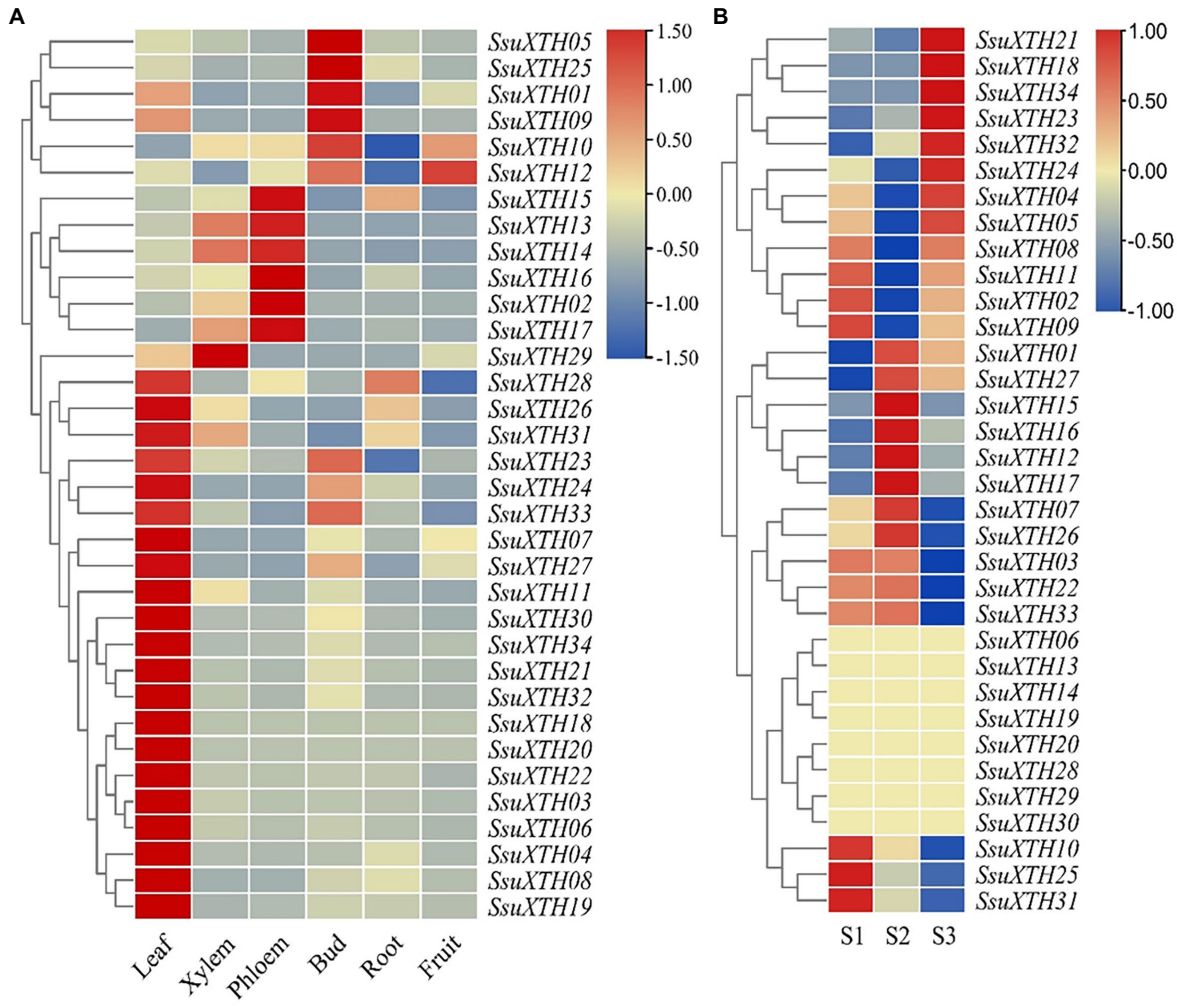
Expression profiles of *SsuXTHs* in different tissues and materials with different vascular characteristics. **(A)** The leaves, phloem, xylem, buds, roots and fruits were collected and then used for qRT-PCR. The expression level was calculated according to the 2^−∆∆Ct^ method. Relative mRNA abundance of each gene was normalized with *SsuACT* gene. **(B)** RNA-seq data of three half-sib progenies (S1, S2 and S3) from SC25 family in *S. superba* with different vascular characteristics were used for expression profiles analysis. The expression level was determined by the FPKM calculated by StringTie v2.1.2 tool and log2 transformation was used for normalization.

The *SsuXTHs* had different expression patterns in different tissues (Figure 5A), which indicated that the *SsuXTHs* gene family might have different functions in developmental processes. The results showed that most *SsuXTHs* (21, 62%) were mainly expressed in leaves. This was followed by the phloem (6, 18%) and buds (5, 14%). Interestingly, the tandem repeat genes (*SsuXTH14*, *SsuXTH15*, *SsuXTH16*, *SsuXTH17* and *SsuXTH29*) were mainly expressed in the phloem and xylem. And the expression of almost all genes in roots and fruits were very low.

### Differential Expression of *SsuXTHs* Between Materials With Different Vascular Characteristics

The expression levels of 34 *SsuXTHs* in S1, S2 and S3 were retrieved from transcriptome databases (SRR18212748 ~ SRR18212756). The results revealed that only 14 differentially expressed *SsuXTH* genes were highly expressed among different materials (Figure 5B; Supplementary Table 10). Among, 10 *SsuXTH* genes were differentially expressed in S2 vs. S1, 12 genes were differentially expressed in S2 vs. S3, and 8 genes were differentially expressed in S1 vs. S3. Only 5 *SsuXTH* genes (*SsuXTH10*, *SsuXTH16*, *SsuXTH17*, *SsuXTH23* and *SsuXTH31*) were differentially expressed in three comparison combinations. In addition, the expression of *SsuXTH12, SsuXTH15*, *SsuXTH16* and *SsuXTH17* in S2 was significantly greater than that in S1 and S3, which was consistent with the fiber length difference, indicating that these genes might positively regulate fiber elongation.

The GO enrichment analysis was conducted on the 14 differentially expressed genes, and it was found that they were involved in 16 GO terms (Supplementary Table 11), including 5 biological processes (BPs), 5 cell components (CCs) and 6 molecular functions (MFs). The BPs mainly included carbohydrate metabolism, polysaccharide metabolism and glucan metabolism, and the MFs mainly included transferase activity and hydrolase activity. The CCs mainly included the cell wall, extracellular region and apoplast, which were basically consistent with the predicted subcellular localizations.

### Functional Interaction Network of SsuXTH Proteins

**Figure 6:**
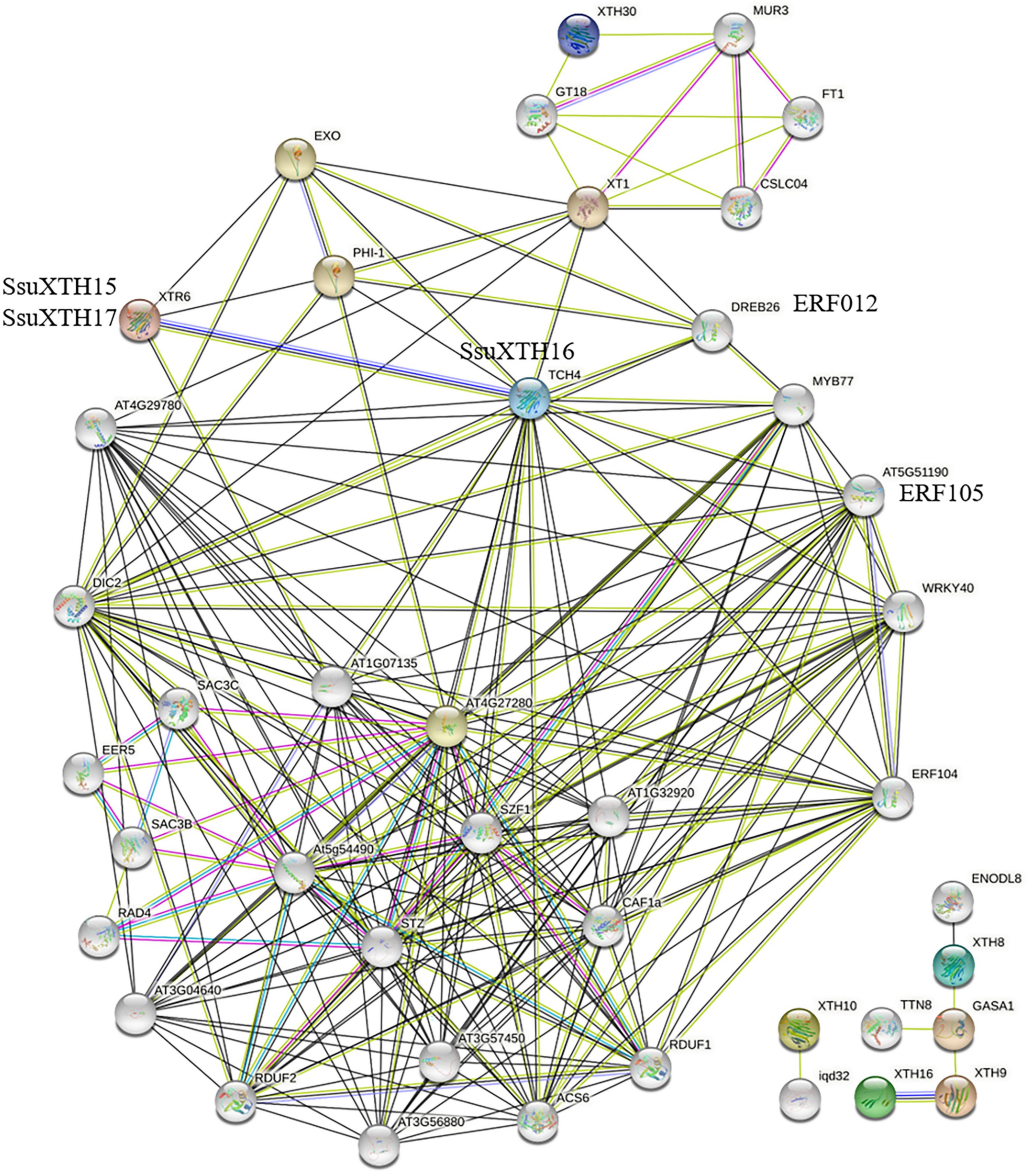
Prediction of the functional interaction network of SsuXTH based on the orthologs in *A. thaliana*. The colored nodes: query proteins and first shell of interactors; White nodes: second shell of interactors; Light blue edges: known interactions from curated databases; Pink edges: known interactions experimentally determined; Green edges: predicted interactions with gene neighborhood; Red edges: predicted interactions with gene fusions; Blue edges: predicted interactions with gene co-occurrence; Blue edges: text mining; Black edges: coexpression; Purple edges: protein homology.

To understand and explore the interaction patterns of *XTH* genes in *S. superba*, a protein interaction network was constructed using the STRING server based on an *Arabidopsis* association model (Figure 6). The *Arabidopsis* model had to be employed due to the absence of the *S. superba* database in the STRING server. The interaction network was therefore constructed mainly based on XTR6 (AT4G25810) and TCH4 (AT5G57560) in *A. thaliana*, the homologs of SsuXTH15, SsuXTH16 and SsuXTH17. The results showed that various transcription factors specifically interacted with SsuXTH16 (Figure 6), such as ethylene-responsive transcription factors (ERF012 (AT1G21910), ERF104 (AT5G61600) and ERF105 (AT5G51190)), MYB77 (AT3G50060), and WRKY40 (AT1G80840). They interacted separately with the phosphate-responsive 1 family proteins PHI-1/EXL1 (AT1G35140) and EXO (AT4G08950). The PHI-1/EXL1 and EXO genes were identified as potential mediators of brassinosteroid (BR)-promoted growth (Schröder et al., 2009), and BR could promote cell expansion by increasing the expression of *AtXTH22* and *AtXTH24* (He et al., 2003). Notably, SsuXTH15/SsuXTH17 and SsuXTH16 could interact with each other.

## Discussion

*XTH* gene families have already been identified and functionally characterized in several plants, including *A. thaliana*, *O. sativa*, *G. hirsutum*, *B. rapa*, *B. oleracea*, *B. juncea* and so on (Yokoyama and Nishitani, 2001; Yokoyama et al., 2004; Lee et al., 2010; Li et al., 2020; Wu et al., 2020). However, the *XTH* genes in *S. superba* have not yet been studied. In this study, we analyzed the gene structure, phylogenetic relationship, genomic distribution, and expression of *XTH* genes in *S. superba* at the genomic level. The results showed that a total of 34 *XTH* genes were in *S. superba*, and they were divided into three groups, which had different gene structures and subcellular locations (Figure 2A). For example, the genes from the ancestral group and Group III were localized in the extracellular space and cell wall, respectively, whereas genes in Group I/II were mainly in the cell wall, cytoplasm, and extracellular space. These findings were similar to those reported in pineapple and poplar (Li et al., 2019; Cheng et al., 2021). Moreover, all the genes in Group III contained three introns and four exons, and the types and numbers of conserved motifs within the group were similar.

Compared with those in *A. thaliana*, *P. trichocarpa*, *E. grandis* and *C. sinensis*, the *XTH* families in *S. superba* were not obviously contracted or expanded (Supplementary Table 3). Segmental and tandem duplication events did not lead to *XTH* gene family expansion but might increase functional divergence, which was an essential factor in adapting to environmental changes (Conant and Wolfe, 2008). Of course, segmental and tandem duplication events also are found among poplar *XTH* family genes (Cheng et al., 2021). Furthermore, the phylogenetic analysis of *XTH* encoded proteins among the five plants revealed that *S. superba* and *C. sinensis*, all belonging to Theaceae, had many more genetic relationships, while *Populus* and *E. grandis* had much closer relationships (Figure 1). In addition, the Ka/Ks ratio indicates different selection pressures and divergence times on genes throughout evolutionary changes. However, in *S. superba*, the Ka/Ks ratios indicated that strong purifying selection pressures had occurred during evolution, thereby enabling a number of different environmental factors to regulate the *XTHs* in the *S. superba* genome. This might also be the reason why most SsuXTH proteins contained similar structures (Zhu et al., 2019). The replication time of most *SsuXTH* gene pairs was between 27.48 and 64.81 Mya, which was consistent with the origin and expansion time of the *Schima* genus (Shi et al., 2017).

*Cis*-acting regulatory elements play the role of key molecular switches in the transcriptional regulation of gene expression (Ding et al., 2018; Verma et al., 2019). *LeXTH2* could be enhanced by GA and inhibited by auxin in tomato (Catala et al., 2001). *AtXTH23* is also upregulated by ABA in *Arabidopsis* (Yokoyama and Nishitani, 2001). These studies show that hormones could regulate *XTH* expression, and the *cis*-acting element in the promoter region of *XTH* is an important factor in the regulatory mechanism (Vissenberg et al., 2005). In this study, we found various phytohormone regulatory elements in the promoter regions of *SsuXTH* genes, including ABREs, AuxREs, GAREs, MeJAREs and SAREs. SsuXTH16 also interacted with ethylene-responsive transcription factors (ERFs) in protein interaction network, and *AtXTH* was upregulated by ethylene in root hairs (Vissenberg et al., 2001). Notably, the promoter region of *SsuXTH* genes contained MYB, MYC, HD-Zip and GATA transcription factor-binding sites, and MYB was fully covered in all *SsuXTHs* (Figure 4). The protein interaction analysis also proved that SsuXTH could be regulated by MYB, as SsuXTH16 interacted with AtMYB77 (Figure 6). Smit et al. (2020) shows that AtMYB77 is a key and core transcription factor in the vascular development transcriptional network and can switch on LBD4 (LATERAL ORGAN BOUNDARIES DOMAIN4), which regulates vascular cell number and organization in *Arabidopsis*. However, there are few reports on transcription factors regulated *XTH* expression. Only ANAC071 is bound to the *AtXTH19* and *AtXTH20* promoters to induce their expression by auxin in the distal part of an incised stem and their involvement in cell proliferation in the tissue reunion process (Pitaksaringkarn et al., 2014). In addition, each *SsuXTH* contained multiple copies of LREs, which were involved in light responsiveness, and *S. superba* had strong light-seeking ability in the early stage (Yao et al., 2018), suggesting that *SsuXTH* genes were an important component of the light response in *S. superba*. Finally, the existence of ARE and WRE suggested that the expression of *SsuXTH* genes might be induced by anoxic conditions and wounds, and this result was consistent with those of Oogawara et al. (2005). *S. superba* could quickly recover vitality after forest fire (Zhou et al., 2020a), and we speculated that anoxic conditions and burns induced the expression of *SsuXTH* genes and then promoted plant regeneration. All these *cis*-acting regulatory elements of *SsuXTH* implied that they had important functions in plant growth, development and stress resistance.

The *SsuXTH* genes had different expression patterns among tissues in *S. superba* (Figure 5A). This indicated that the *XTH* gene family provided opportunities to break the functional constraints of the original gene during the course of evolution. The same patterns were also found in barley and tobacco (Wang et al., 2018; Fu et al., 2019). And 5 *SsuXTH* genes (*SsuXTH10*, *SsuXTH16*, *SsuXTH17*, *SsuXTH23* and *SsuXTH31*) were differentially expressed in three comparison combinations among different materials (Figure 5B), but only *SsuXTH16* and *SsuXTH17* were mainly expressed in the phloem and xylem. It was interesting that we found that most of the tandem repeat genes were highly expressed in the phloem and xylem, and from the phenotype correlation results, *SsuXTH15*, *SsuXTH16* and *SsuXTH17* might positively regulate fiber elongation (Figure 5B). The GO enrichment analysis showed that 14 differentially expressed genes were involved in carbohydrate metabolic processes and possessed transferase and hydrolase activity (Supplementary Table 11), which indicated that the SsuXTH enzyme mainly participated in glycoside metabolic processes by transferring and hydrolyzing xyloglucan in the cell wall and then regulating fiber elongation.

## Conclusion

This study systematically identified and characterized the *XTH* family in *S. superba*. A total of 34 *SsuXTHs* were obtained, which were classified into three subfamilies based on the phylogenetic relationship and unevenly distributed on 18 chromosomes. Furthermore, the intron–exon structure and conserved motif composition of them supported the classification and the members belonging to the same subfamily shared similar gene structures. Segmental and tandem duplication events did not lead to *SsuXTH* gene family expansion, and strong purifying selection pressures during evolution led to similar structure and function of *SsuXTH* gene family. The interaction network and *cis*-acting regulatory elements analysis provided useful clues for revealing the *SsuXTH* regulation pathway. Finally, expression profiles and GO enrichment analysis showed most of the tandem repeat genes were mainly expressed in the phloem and xylem and they mainly participated in glycoside metabolic processes through the transfer and hydrolysis of xyloglucan in the cell wall and then regulated fiber elongation. Here, we provide a new insight into the function of *SsuXTH* and lay the foundation for further exploration of the decisive genes of fiber development. However, most of the analyses were primarily *in silico*, next we would identify the function of tandem repeat *SsuXTH* genes through genetic transformation and *in vitro* enzyme activity etc.

## Data Availability Statement

The datasets presented in this study can be found in online repositories. The RNA-seq data in this study were deposited in the NCBI Sequence Read Archive (SRA) database under numbers SRR18212748 ~ SRR18212756.

## Author Contributions

ZY, RZ, and ZZ designed the research. ZY conducted the experiment, analyzed the data, and wrote the manuscript. All authors contributed to the discussion of the results, reviewed the manuscript, and approved the final article.

## Funding

This study was supported by the Zhejiang Science and Technology Major Program on Agricultural New Variety Breeding (grant number 202102070–9), the Science and Technology Innovation Project in Jiangxi Province (grant number 2019–19), and the Sixth Stages of Planting and Seedling Science and Technology of Fujian Province (grant number 2019–06).

## Conflict of Interest

The authors declare that the research was conducted in the absence of any commercial or financial relationships that could be construed as a potential conflict of interest.

## Publisher’s Note

All claims expressed in this article are solely those of the authors and do not necessarily represent those of their affiliated organizations, or those of the publisher, the editors and the reviewers. Any product that may be evaluated in this article, or claim that may be made by its manufacturer, is not guaranteed or endorsed by the publisher.

## Footnotes

^1^ http://tpia.teaplant.org/

^2^ https://phytozome-next.jgi.doe.gov/info/Ptrichocarpa_v4_1

^3^ https://phytozome-next.jgi.doe.gov/info/Egrandis_v2_0

^4^ https://www.arabidopsis.org/

^5^ https://pfam.xfam.org/

^6^ http://www.ncbi.nlm.nih.gov/Structure/cdd/wrpsb.cgi

^7^ http://web.expasy.org/protparam

^8^ http://www.csbio.sjtu.edu.cn/bioinf/euk-multi2/

^9^ https://itol.embl.de/

^10^ http://meme.nbcr.net/meme/cgi-bin/meme.cgi

^11^ http://rice.uga.edu/

^12^ http://bioinformatics.psb.ugent.be/webtools/plantcare/html/

^13^ https://string-db.org/?tdsourcetag=s_pctim_aiom

^14^ https://services.healthtech.dtu.dk/service.php?TMHMM-2.0

^15^ https://services.healthtech.dtu.dk/service.php?SignalP-5.0

^16^ https://services.healthtech.dtu.dk/service.php?NetPhos-3.1

^17^ https://npsa-prabi.ibcp.fr/cgi-bin/npsa_automat.pl?page=npsa_sopma.html

^18^ https://swissmodel.expasy.org/
