# Supplementary material for: The XTH Gene Family in Schima superba: Genome-Wide Identification, Expression Profiles, and Functional Interaction Network Analysis
Source: Front Plant Sci. 2022 Jun 16;13:911761. doi: 10.3389/fpls.2022.911761 (PMC9243642; doi:10.3389/fpls.2022.911761)
Supplement: Supplementary file 1 [file Data_Sheet_1.ZIP › Supplementary/Supplementary Table 2.docx]

**CDS sequence**

>SsuXTH01

ATGGCCACATTAGCATACCCATCCTTTGTTGTTTCTCTTTTCATACACGTCCTTGCCTTTTCCCTCTCGGTTTCCGGTCGACCCGCCACTTTTCTTCAAGACTTTAGAATCACTTGGTCCGATTCTCATATCAGGCAAATTGATGGAGGGAGGGCCATACAACTCGTTCTGGACCAAAATTCAGGTTGTGGTTTTGCATCCAAGAGTCAATACTTGTTCGGACGTGTTAGCATGAAGATCAAGCTCATTCCGGGGGACTCTGCCGGCACTGTCACCGCCTTTTATATGAATTCGGACACCGACACTGTACGTGACGAGCTGGATTTTGAATTTTTGGGGAATCGGACCGGACAACCTTATACTGTCCAAACCAATGTCTATGCCCATGGGAAGGGCAATAGGGAACAGAGGGTGAACCTTTGGTTCGACCCTGCCGCAGACTTTCACACTTACTCAATACTGTGGAACCATCATCATGTTGTGTTTTATGTGGATCAAGTACCTATAAGGGTGTACAAGAACAATGAAGCAAGAGGACTCCCGTACCCAAAGCTCCAACCCATGGGAGTCTACTCAACACTGTGGGAAGCCGATGACTGGGCAACAAGAGGTGGGATCGAGAAGATCGATTGGAGCAAAGCCCCATTCTATGCCTACTACAAGGACTTCGACATAGAAGGATGCCCAGTTCCAGGACCCACAACTTGTGCCTCAAACTCAGCAAATTGGTGGGAAGGAACTACTTATCAACAGCTCAGTCCCACCGAAGCTAGAAGCTACAAGTGGGTTCGCATGAATCACATGATCTACGACTACTGTGTCGACAAATCACGATATCCAGTTACCCCACCAGAATGTGTGGCAGGGATTTAA

>SsuXTH02

ATGTGTGAAATGACAAATATTCTTCATATAGAAAATATCCAAAGTCAGAGACAGCATAAGAAGAAGAAGAGGAAGAAGGTGGAGAAAGGTTTACCTGATGAGAGCTCTCAAAGGAGGAAAGAAGGGAATGAAAACGATAATCGCAATAGCAGAAAGGGGCCAAAGAGGTCAACGCCACTGGCGAGAATATTTACATCAAACTCTATTGGAGTTGAACAGCAACTTGTGGTGAATAACCATGGTGATGGAGAGCGGCAAGTGAGGGTTGGGAAGGGGTGGGGATGGGTTGGGGAGGATTGTCAGTGGGTAGAGGGAGGTCCGGAGTTGCTTCTCCGGAGCTGCCGTTTTTCTGTGGATGGCACACCCATTAGAGAGTTCAAGAATGCAGAGTCAATTGGAGTTCCATATCCAAAGAACCAACCCATGAGGATATACTCCAGTCTCTGGAATGCTGATGACTGGGCAACAAGAGGTGGGCTCGTCAAGACTGACTGGACACAAGCACCATTCACCTCCTCGTACAGAAACTTCAACGCGGACGCTTGCATTTGGTCATCTGGATCATCTTCTTGCAGTTCGAGTAGTACTCCATCTTCAACCTCTGGCACTGCCAATTCATGGCTCTCTCAAGAGTTGGACTCCGCAAGCCAAGACAGGCTGAGATGGGTGCAGAAGAACTACATGATTTACAATTACTGCAACGACACCAACAGATTTCCCAAGGGCCTTCCATTAGAATGCAGCATGTCTTAG

>SsuXTH03

ATGGCAAGTTCACAAGCACTGCTCCTAGCTATATTCATATCAGCAATTGCATTTCATTCAAGATCAGTAGATGCAAACTTTGGTGAGAGCATGTATTTCAACTGGGGTGCTCACCATTCTTGGATGGGAAACAACGGGAAAGATCTTCAACTTGTTTTGGATCAATCTTCAGGTTCTGGTGTCCAAACAAAGAATGCATATCTATTTGGAAGCATTGAAATGCTAATCAAGTTGGTACCTGGGAACTCTGCCGGAACTGTCACAGCCTATTATATGTCCTCGACTGGAGACAAGCATGACGAGATAGACTTTGAGTTCTTGGGTAACTCATCGGGACAACCTTACATTATCCATACAAACATCTTTACGCTAGGAATTGGAAACAGAGAACAGCAATTCCACCCCTGGTTTGACCCAACAGCTGATTTCCACAACTACACCATACATTGGAACCCAACAGAAGTTGTGTGGTATGTTGATAGTGTGGCTATTCGAGTATTCAGAAACTACCAGAGCGAGGGGATTCCCTACCCAAACGAACAAGGGATGAGGGTTTACTCCAGCTTATGGAATGCTGATAACTGGGCGACAAGAGGTGGCCTTGTTAAGATCAATTGGAACAGTGCACCTTTGATAGCCAGATACCGCAATTTCAGGGCAAGGGCTTGCAAGTGGAATGGGCCAATTAGCATTAGTCAATGCGCCTTCCAGACGCCTGCCAACTGGTGGACCTCCCCAACATACAGCCAATTGAGCTATGCTAAGCAGGGTCAGATGAAGTGGGTCAGAGATAACTACATGATCTATGACTACTGCAAAGATACTAAGCGATTCAATGGACAGATGCCACCTGAATGTTTCAAACCACAATACTAA

>SsuXTH04

ATGGATAGTTTTCCTTCTCTCTCCAAAGCATGTCTCTCTTTCCTTCTCCTCCTTCTCCATCTTCTCTCTGCTACTGCTAACGCTGCCTTCAACGTACCCACAATTTCATTCAACAAAGGCTTCAATCCTCTCTTCGGCCACAGCAACCTCATCCGATCATCCGACGACAAAAGCGTCGGTCTCGTCCTCAACCGATACACAGGTTCTGGGTTCAAGTCTTCCGAGCTCTACAACCATGGCTTCTTCAGTGCTAAAATAAAGTTGCCATCTGAATACACTGCTGGCGTAGTCGTTGCCTTCTATACGTCGAACGGAGACATATTTGAGAAAACACACGATGAATTGGACTTTGAATTTCTGGGAAACATTAGAGGAAAGCGATGGAGGTTTCAGACAAACGTGTATGGAAATGGAAGCACAAGTAGAGGAAGAGAAGAGAGATACACACTGTGGTTTGACCCCTCCAAAGAGTTCCATCGTTACAGCATTCTGTGGACCAACAACATCATCATCTTTTACATAGACGATATTCCGATTCGCGAGATAGTACGAAGCGATGCCATGGCCGGAGACTTCCCCTCAAAACCAATGTCATTGTATGCCACCATATGGGATGCTTCGAATTGGGCTACCTCCGGTGGCAAATACAAGGTCAACTACAAGTATGCTCCATTCGTGGCCAAATTCACCGACTTCTCCCTCCACGGATGTGCTTCTGATCCTATTCAAGAGGTGTTATCCTCTAGCTGCTCTCAAAAAGATGATCAATTAGAAAGCACAAACTATGCTCATATCGCGCCAAAACAACGAGTGGCCATGAAAAAGTTTAGGCAAAAATACATGTACTATTCATACTGCTATGACACGGTAAGGTATCCTATTCCACCACCAGAATGTGTGATTGATCCATCAATGAGGCAGCAGTTTAAGGATACTGGGAGGTTGAAGTTTGACGAGAGGCGGCGCCACCACTCCAAGAGAAGAAACCAGGTTTCAAGCATGAAGAACTATGGAGACCAGGATGAGGATTGA

>SsuXTH05

ATGTGCATATTGCCTTCAATCAATCATCCTTTGCAGCAGAATTCAATAAAAACAAAAATGATGAAATATCTGTGTCTTTTAGCCATTATCAGTGTTCTTGCCACGCATTTGGTTCAGACTTCAGAAGCTTCTCTGGTATCGAAAGGAGATTTCGATAAGGACTTCTTGGTCATATGGTCTCCTAGCCATGTAAATACTTCTGCAGATGGCTTAGTGAGAAGCTTAAAGCTGGACAATGACTCAGGCTCAGGCTTTGCCTCGAATGATACATTCCTATTTGGTCAGTTTGACATGCAAATTAAGCTGATAGAAGGTGACTCTGCTGGCACAGTGGTGGCCTTTTATCTCCAATCTAATCAATCCAATGGTGATAATGAGATGGACTTTGAATTCCTTGGAAATGTCATTGGACAGCCATATATACTTCAAACCAATGTTATTCTTGATGGGAATGGAAACAGGGAAGAGAGAATTCACTTGTGGTTTGATCCAACAAAGGACTTCCACACTTACTCCATATTATGGAGCCTTCACCAAATTGTATTCATGGTGGATTGGGTTCCCATAAGAACATACAGAAACTACACAGACAAGGGAGTGTCATTTCCACAGTGGCAGCCAATGAGACTTTTAATCAGCCTATGGGATGGCGACAGCTGGGCAACGAGAGGCGGCCGCGACAAGATTGACTGGTCAAAGGGACCCTTCATAGCCTCATTCAAGAATTACAAAATTGATGCATGTGTGTGGAATGGAGATGCAAAATTTTGTAGGGAAAACAACTCTAGTAATTGGTGGCACAATGACAGGTTCAGCACTCTAACAGGGACACAGAGGAGGTTATTCAAGTGGGTTAGGAAGTACCACCTCATATATGATTATTGTCAAGACAATGTTAGATTCCAAAACCAACTTCCCATGGAATGTTATCTTTCTAAGTACTGA

>SsuXTH06

ATGGGCTTGCAATCAAGGAACACAAACCCCTCTAGGTCCTCCTCTTTCCTTCCCCCTGGTTGCGTCCCTATCCTCGTTTTCTTCTCCGTCTTCGCCTTTTTCTTCCTCTTCAACCTCGACACCTTCCTGTCTCGTACCAAAGCCATTGCGCGCCATAGTTTCAATGAACAAACCCGCTTGTTCATGAATGCCGAGGGTGGCAACCTTGAAAGAGAATTTGACATCACGTGGGGCGATGGGCGTGCCAAAATACACAACAATGGTGGGCTTCTGACTCTGTCACTCGACAAAAACTCAGGCTCGGGGTTTCGATCCAAAAACGAGTACTTATTTGGAAAGATTGAGATGCAAATCAAGCTAATATCCGGGAACTCAGCTGGCACAGTCACTACCTACTATTTGTCATCGGAAGGGCCAACCCATGATGAGATAGATTTTGAGTTTTTAGGGAATTCAAGTGGTGACCCTTACACTCTTCACACCAATGTGTTTTGTCAAGGCAAAGGCAACAGAGAACAACAATTTTTTTTGTGGTTTGACCCAACTTTGGATTTTCATACCTATTCCATACTTTGGAACCCCTATAGAATTGTATTCTATGTGGATGGAACACCCATTAGAGAGTTCAAGAACTTGGAGTCAAAGGGTATTCCATATCTAAAGGACCAACCCATGAGAATATACTCTAGCCTATGGAATGCTGATGATTGGGCAACAAAAGGTGGGCTTGTCAAGACTGATTGGAAAAAAGCTCCTTTCACTGCTTCGTATCGTAACTACAAACCTAATGCTTGCGTTTGGTCTGAAGGGACATCATCTTGTAGTTCAAGTTCCCTAACTTCTAACTCGACTAATTATTCATGGATGTCGGAAAAGCTCAACTCTACAGGCCTTGAGAGAATGAAATGGGTGCAGAAGAATCACATGGTGTACAATTACTGTATAGACAAGATGCGATTTCCAGAGGGCCTTGCTCCAGAATGCAAGCTGTCTTAG

>SsuXTH07

ATGGAGAGAGGGTTCTCTCCAATGGCAACTCTTCTCTGCATTGCAGTTCTTATAGCTGCTTATTGTTTATCAGTCTCCCAATCTCAGTCAACAACTCCATTCCAAGACAATTTCAGTATAATGTGGTCTGAAAACCATTTTAAAACCTCTCCGGATGGGCAGATCTGGTATCTCTCTTTGGACAACGACACAGGTTGTGGGTTTCAAACAAAGCAGAGATACAGATTTGGGTGGTTCAGCATGAAGCTCAAGTTGGTCGGAGGTGACTCTGCTGGAGTTGTGACAGCTTATTATATGTGCTCGGAGAATGGGGCGGGGCCAGAGAGAGATGAGGTGGATATTGAGTTTTTGGGGAACAAAACAGGGCAGCCATACCTGATACAGACAAACGTCTACAAGAATGGAACTGGCAACCGTGAGATGAGGCACATGCTTTGGTTTGATCCCACTGAGGATTTCCACTCCTATTCTATCCTCTGGAACACCCACCAGCTTGTGTTCTTTGTGGATCAAGTTCCAATTAGGGTGTACAAGAATGCAAATTATACAAACAACTTCTTCCCAACTGAGAAGCCAATGTACCTGTTCTCAAGCATATGGGATGCAGATAACTGGGCGACGAGAGGCGGGCTCGACAAGACAAATTGGACTAATGCACCATTTGTTTCTTCTTACACGGACTTCAGTGTTGATGCTTGCCAGTGGGTGGACCCTTACCCAGCATGTGTATCCACCACCACCAACAACTGGTGGGATCAGTATGCTGCTTGGCACCTCTCTGATCAACAGAAGACTGATTTTGCTTGGGTTGAGAGGAACCTTGTTGTTTATGATTATTGCCAGGACAACAAAAGGTTCCCAACAATCCCTGAGGAGTGCTCACTAAGTCCATGGGATTGA

>SsuXTH08

ATGGTGATTTGCATATCTTGTAAAGCTTCAGTGATGCTGTTAGCATCCCTAATTGTGAGTTGTTCATTCATCGTTGCCTCTGCCAGTAACTTCTACGACGACTTTGATATAACGTGGGGTGATCAACGAGCCAAAATACGCAATGGCGGCCAGCTTCTGACTCTCTCTCTTGACAACGCCTCTGGCTCCGGCTTTCAGTCCAAGAGAGAATACCTCTTCGGAAGGATCGACATGCAACTCAAGCTTGTCTCTGGTAATTCTGCTGGCACTGTCACTGCTTACTATTTGTCTTCTCAAGGCTCAACCCATGACGAGATTGATTTTGAGTTCTTGGGAAATGTTAGCGGGCAACCCTATATTGTTCACACTAATGTGTTCAGTCAAGGGAAAGGGAATAGGGAGCAACAATTCTATCTCTGGTTTGATCCTACAAGAAATTTTCACACCTATTCCGTTGTTTGGAACCCCCAAAACATTATCTTCATGGTAGATAATAAACCCATAAGAGTTTTCAAAAACGAAGAGGCCACAACTGGAGTCCCGTACCCTAAAAACCAACCCATGAGAATATACTCGAGCCTCTGGAACGCTGATGATTGGGCAACACAAGGAGGTCTTGTCAAAACCGACTGGTCCAAAGCTCCATTCACTGCTTTTTACAGAAACTTCAAAGCTGACGCTTGTGTACCATCTTCTTCTTCTTCATGCTCTTCCCTGTTCACCACGAGTTCCGTGTCTGGCGGTGCTGCCGCGGAGTGGCAAACTCAGGGGCTCGACGCAGCGGGTCGAAAGAGACTTAGGTGGGCCCAAAAGTATTACATGGTTTACAACTACTGCAGCGATTTGAAGCGTTTTCCACAGGGCCTCCCACCTGAATGCACACGCTAA

>SsuXTH09

ATGGGTGCTGCACCCAGGAAGCCTGTGGATGTTCCCTTTGGTAGAAACTATGCTCCTACTTGGGCTTTCGATCACATTAAGTACTTCAATGGAGGCTCTGAGATTCAGCTCTACCTCGATAAGTACACTGGTACTGGGTTCCAGTCGAAGGGATCTTACTTATTCGGCCACTTCAGTATGCAAATAAAGATGGTTCCAGGAGATTCTGCAGGGACAGTGACTGCTTTCTATCTATCATCTCAAAACTCGGAGCATGATGAAATAGACTTTGAGTTCTTGGGGAACAGGACAGGCCAACCCTACATATTGCAGACAAATGTGTTCACAGGAGGGAAGGGGGACAGAGAACAGAGAATTTATCTGTGGTTCGACCCAACTAAAGAATACCACTCTTACTCTGTTTTGTGGAATCTCTACCAGATTGTATTCTTCGTGGACGATGTACCAATCAGGGTGTTCAAGAACTGTAAAGATTTGGGAGTGAGGTTTCCATTCAATCAGCCAATGAAGATATACTCGAGCCTCTGGAACGCCGACGACTGGGCCACAAGGGGCGGGCTCGAGAAGACCGACTGGTCTAAGGCTCCGTTCGTGGCGTCCTACAAGAGCTTTCACGTGGACGGCTGCGAGGCCTCCGTCGAAGCCAAGTTCTGCGACACACAGGGAAAGCGCTGGTGGGACCAGAAGGAATTCCAAGACCTCGACCCTGCTCAGTACCGGAGGCTCCAATGGGTCCGCCAAAAGTACACCATCTACAACTACTGCTCCGATAGAACCAGATACTCCACCATCCCCCCTGAATGCAAAAGAGACCGAGACATTTAA

>SsuXTH10

ATGGCTTTCTTTCTTCCACTTCTCCTCATTCTTATGGTCCCTTCAACAACCCATGCTTCCTATTGGCCACCCTCCCCTGGCTACTACCCAAGCACCAAGTTCAGGTCTATGACCTTTTACCAAGGTTTTAGAAACCTCTGGGGTCCTCAGCATCAAAGAATGGACAGCAATGCTTTAGCAATCTGGCTTGATAGCACCTCAGGAAGTGGGTTCAAGTCTGTTCGCCCGTTTCGATCCGGGTATTTTGGTGCCAACATCAAGCTGCAACCTGGTTACACTGCAGGAGTTATTACAGCTTTTTATCTTTCAAACAATGAAGCTCATCCAGGGTACCATGATGAGGTGGACATTGAATTCTTGGGGACAACATTTGGGAAGCCATATACACTGCAGACAAATGTTTACATCCAAGGAAGTGGGGATGGGAGGATTATAGGGAGAGAGATGAAGTTTCACCTATGGTTTGATCCTACAAAAGCTTTTCATCACTATGCCATACTATGGAGTCCTAGAGAGCTTATATTCCTTGTGGATGATGTGCCAATAAGGAGGTACCCAAAGAAGAGCGTGGCAACATTTCCACTAAGGCCAATGTGGCTTTATGGCTCCATATGGGATGCCTCTTCCTGGGCTACAGAGGAAGGTAAATACAAGGCTGATTATAGGTACCAACCATTTGTTGGAATGTACAACAATTTTAAAGCAACTGGTTGCTCAGCCTATGCTCCGCGGTGGTGCCACCCGGTCTCTGCCTCCCCCTACCGGTCTGGGGATCTCACCAGAAGGCAGTACATGACCATGAGATGGGTTCAAACCCACTCCTTGGTGTATGACTATTGCAAGGACCCGAAGAGGGACCATTCCCTAACTCCTGAATGTTGGGGTTAA

>SsuXTH11

ATGGCCTTTTCCAATAATGGGTTCTCTTTCTCCATGCTATTGTTGGTTATTCTAATGGGGATGAGCTCTTTGATGGTTGGCTATGGTGGCAACTTCTACCAAGACTTCGACATAACATGGGGGGGTAGTAATCAACGAGCCAAGATGTTCAATGGAGGTCAGCTTCTCACCCTGTCCTTAGACAAGATCTCTGGTTCTGGTTTTAGATCCAAGAAAGACTACTTGTTTGGGAGGATTGATATGCAGCTCAAACTTGTTTCTGGCAACTCTGCTGGCACTGTTACTGCCTACTATCTGTCTTCTGAAGGGCCAACACACGATGAGATTGATTTCGAGTTCTTGGGAAACCTCAGTGGTGACCCTTACATCCTCCACACCAATGTCTTCACTCAAGGCAAAGGGAACAGAGAGCAACAGTTCTATCTTTGGTTCGACCCCACCAAAAATTTCCACACTTACTCGATCATCTGGAACCCACATAATATCATTTTCATGGTGGATTACACTCCCATAAGAGTATTCAAGAATGGTGAATCAATTGGTGTCCCTTTCCCCAAAAACCAACCCATGAAGATCTACTCTAGTCTCTGGAATGCCGATGACTGGGCCACCAGAGGCGGGTTAGTGAAAACCGATTGGACAAAAGCACCGTTTACAGCGTACTACCGGAACTTCAATGTTCAAACCTGTTCAGGGACATGCACTTCTTCTAGTACTTCTTCATTGTCGAATGGGGCATGGCAGAGCCAGGAACTTGATGCCTACAGTCGAAGACGACTGAGATGGGTTCAAAAGAATTTTATGATTTACAATTACTGCACAGATCGTAGACGTTTCCCCCAAGGCCTTCCTCCAGAGTGTAGGCAACGAAGGTTCCTATGA

>SsuXTH12

ATGGCTTTCTTTCTCTCACTTCTTCTCATTCTTTTTTTCTCTTCAAGCAATGCTGCATATTGGCCACCCTCACCTGGGTACTACCCAAGTTCCAAGTTTAGGTCTATGACTTTTTACCAAGGCTTTAGAAACCTTTGGGGTCCTGAGCATCAAAGACTGGACAACAATGCATTGACAATCTGGCTTGATAGGACCTCAGGAAGTGGGTTCAAGTCGGTTCGCCCGTTTCGATCTGGGTATTTTGGTGCTTCCATCAAGTTGCAGCCTGGTTACACTGCAGGAGTTATAACGGCTTTCTATCTTTCGAACAATGAAGCTCATCCAGGGTACCATGATGAGGTGGACATCGAATTCCTTGGGACTACATTTGGGAAACCATACACATTGCAGACTAATGTTTACATCAGTGGAAGTGGCGATGGGAAAATCATAGGGAGAGAGATGAAGTTTCATCTATGGTTTGATCCCACAAAAGGTTTCCATCACTATGCCATCCTGTGGAGCCCCAAGGAGATCATATTCCTTGTGGATGATGTGCCCATAAGGAGGTACCCTAGGAAGAGTGTGGCAACTTTCCCACTAAGGCCAATGTGGGTCTATGGATCTATATGGGATGCCTCATCTTGGGCCACAGAGGATGGAAGATACAAGGCAGATTATAGGTACCAACCATTTGTTGGAATGTACAACAACTTCAAAGCCACTGGTTGCTCAGCCTATGCTCCCGCGTGGTGCCGCCCGGTCTCCGCCTCCCCGTTCCGGTCCGGAGGTCTTAGCAGAGGGCAACACATGGCCATGAGATGGGTCCAAACCCACTACTTGGTTTACAACTATTGCAGGGACCCCAAGAGGGACCATTCCCTGACACCAGAATGTTGGGGTTAA

>SsuXTH13

ATGAGGATATACTCTAGCATATGGAGTGTTGATGACTGGGCAACAAGGGGAGGACTTGTGAAGACTGACTGGACTAAAGCTCCCTTCACAGCTTCATACGAAAACTTCAATGCCAATGCTTGCATTTGGTCTTCTGGATCATCTTCTTGCAATTCAAGTTCTTCTAAAAGCAATGCATGGTTCTCAGAACAGTTGGACTCCACAAAGCAAGGGAGAGAGTTCAAGAATGCAGAGGCAGTTGGTGTTCCCTACCCAAAGAACCAACCCATGAGGATATACTCGAGTTTGTGGAATGCTGATGACTGGGCAACAAGGGGAGGGCTTGTGAAGACTGACTGGACTAAAGCTCCCTTCACAGCTTCATACAGAAATTTCAATGCCAATGCTTGCATTTGGTCTTCTGGATCATCTTCTTGCAGTTCAAGTTCTTCTAAAAGCAATGCATGGCTCTCAGAGCAGTTAGACTCCACAAACCAAGGGAGGTTGAAATGGGTGCAGAAGAACTACATGATATACAATTACTGCACTGATTCAAAGAGATTTCCTCAGGGATTCCCTCCTGAATGCAGCATGTCCTAG

>SsuXTH14

ATGAGGATATACTCTAGCATATGGAATGCTGATGACTGGGCAACAAGGGGAGGACTTGTGAAGACTGACTGGACTAAAGCTCCCTTCACAGCTTCATACGAAAACTTCAATGCCAATGCTTGCATTTGGTCTTTTGGATCATCTTCTTGCAATTCAAGTTCTTCTAAAACCAATGCATGGTTCTCAGAACAGTTGGACTCCACAAACCAAGGGAGAGAGTTCAAGAATGCAGAGTCAGTTGGTGTTCCCTACCCAAAGAACCAACCCATGAGGATATACTCGAGTTTGTGGAATGCTGATGATTGGGCAACAAGGGGAGGGCTTGTGAAGACTGACTGGACTAAAGCTCCCTTCACAGCTTCGTACAAAAACTTCAATGCCAATGCTTGCATTTGGTCTTCTGGATCATCTTCTTGCAATTCAAGTTCTTCTAAAATCAATGCATGGTTCTCAGAACAGTTGGACTCCACAAACCAAGGGAGATTGAAATGGGTGCAGAAGAACTACATGATATACAATTACTGCACCGACTCAAAGAGATTTCCTCAGGGATTCCCTCCTGAATGCAACATGTCCTAG

>SsuXTH15

ATGGCTTCTTCTCCTTCTTCTTCTTGTAGCTTAGTACTGTTGATGATATCTGCAATTATATTCAGCTCTGTATTGGCTATTGTCTCAGCTGGTAACTTCAATCAAGACTTTACCATCACATGGGGAGATGGCAGGGCTAAGATCCTCAATAATAGCCAACTTCTTACGCTGTCGCTCGACAAAACCTCCGGCTCAGGCTTCCAGTCCAACAATGAATATCTCTTTGGAAAGATTGATATGCAGCTCAAACTTGTCCCTGGAAACTCTGCTGGCACTGTCACCGCCTACTATTTATCATCACAAGGATCAACCCATGATGAGATAGACTTTGAATTCTTAGGGAATTTGAGTGGTGATCCTTACATTCTTCACACCAATGTGTTTAGCCAAGGTAAAGGCAACAGAGAGCAACAATTCTATCTATGGTTCGACCCCACTGCTGATTTCCATACCTACTCCATCCTCTGGAACCCACAACGCATCATCTTCTCTGTAGATGGCACACCCATTAGAGAATTCAAAAATGCAGAGTCAGTCGGTGTTCCATACCCAAAGAACCAACCCATGAGGATATATTCAAGTCTGTGGAATGCTGATGATTGGGCAACAAGAGGAGGGCTTGTGAAGACTGATTGGACTAAAGCTCCCTTCACAGCTTCATACAGAAACTTCAATGCCAATGCTTGCATTTGGTCTTCTGGATCATCTTCTTGCAGTTCAAGTTCTTCTAAAAGTAATACATGGTTCTCAGAACAGTTGGACTCCACAAACCAAGGGAGGTTGAAATGGGTGCAAAAGAACTACATGATATACAATTACTGCACCGACTCAAAGAGATTTCCTCAGGGATTCCCTCCTGAATGCAGCATGTCCTAG

>SsuXTH16

ATGGCTGCTTCTTCTTCTTCTTCTTCTTCTTCTTCTTCTTCTTCTTCTTCTTGTAGTTCAGTATTGTTGATGATATCTGCAATTGTCTTCAGCTCTGTATTGGCTGTTGTCTCAGCTGGTAACTTCAATCAAGACTTTACCCTCACATGGGGCGATGGCAGGGCTAAGATTCTCAACAATAGCCAACTTCTCACTCTCTCACTCGACAAAACCTCTGGCTCAGGCTTCCAATCCAACAGCGAATATCTCTTTGGAAAGATTGATATGCAGCTCAAGCTTGTCCCCGGAAACTCTGCTGGCACTGTCACCGCCTACTATTTGTCATCACAAGGATCAACCCATGATGAGATAGACTTTGAATTCTTGGGGAATTTGAGTGGTGATCCTTACATTCTTCACACTAATGTGTTTAGCCAAGGCAAAGGCAACAGAGAACAACAATTCTACCTATGGTTTGACCCCACTGCCGATTTCCACACCTACTCCATCCTTTGGAACCCCCAACGCATCATCTTCTCTGTAGATGGAACACCCATTAGAGAGTTCAAGAATGCAGAGTCAGTTGGAGTTCCATACCCAAAGAATCAACCCATGAGGATATACTCTAGTCTTTGGAATGCTGATGATTGGGCAACAAGGGGAGGACTTGTCAAGACTGATTGGACACAAGCACCCTTCACTGCTTCATACAGAAATTTCAATGCCAATGCTTGCATTTGGTCTTCTGGATCATCTTCTTGCAGTTCAAGTTCTTCCTCATCTTCTAAAAGTAATGCATGGCTCTCAGAACAGTTGGACTCCACAAACCAAGGGAGGTTGAAATGGGTGCAGAAGAACTACATGATATACAATTACTGCAATGATTCAAAGAGATTTCCTCAGGGATTCCCTCCAGAATGCAGCATGTCCTAGAAAATTAAAATATATCACTTCCTTTCTCATAGTCATACCCATGAAGTTTTGCTGTACTTTCTTTTTTTAATGTAAATTCCACTATTCAATTCTTTTATTTTTTTTCTCATTGTAATCGTTCATGAAGCCAATAAAATAAACAGTTCTTTTCTATGCTCATCCACAACTAACGTTTGGGTCTCTGATTAA

>SsuXTH17

ATGGCTTCTTCTTCTTCTTCAAGAGTTTCATCAGTGTTGTTGCTAATGATGTCTCTTGTAGTAGGCAGTTTATTCATTGTGGGCTCAGCTAGCAATTTCAATCAAGATTTTGACATCACTTGGGGCAATGGTCGTGCTAAGATACTCAACAATGGAAATCTTCTTACTCTCTCACTTGACAAACCCTCCGGCTCTGGCTTCCAGTCCAAGAACGAGTATCTCTTCGGCAAGATTGACATGCAGCTCAAACTCGTTCCCGGAAATTCAGCTGGCACTGTCACCGCTTACTATTTGTCTTCACAAGGATCTACCCACGATGAGATTGACTTCGAGTTCTTGGGGAATTTGAGTGGTGATCCTTACATTCTTCACACCAATGTGTTTAGCCAAGGCAAAGGCAACAGAGAACAACAATTCTACCTATGGTTCGACCCCACTGCTGATTTCCACACCTACTCCATCCTCTGGAACCCCCAACGCATCATCTTCTCTGTAGATAGCACACCCATTAGAGAGTTCAAGAATGCAGAGTCAATCGGTGTTCCCTACCCAAAGAACCAACGCATGAGGTTATACTCCAGTTTGTGGAATGCTGATGACTGGGCAACAAGAGGTGGACTCGTCAAGACTGACTGGACACAAGCTCCTTTTACTGCTTCATACAGAAACTTCAATGCCAATGCTTGCATTTGGTCTTCTGGATCATCTTCTTGCGGTTCAAGTACTCCCTCTTCATCTTCTAATAGCAATGCATGGTTCTCAGAACAGTTAGATTCCACAGGCCAAGAGAGATTGAAATGGGTGCAGAAGAACTACATGATATACAACTATTGCTCCGACGTAAAGAGATTTCCTCAAGGTTTCCCACCGGAGTGCAACATATCCTAG

>SsuXTH18

ATGGAGAAGAAGGGGTACGAAGTTGCAAATCTAGAAAGGCAGATGAGGAAAGGCAACGAGAAGATGAGAGTCTACAACTTCACTCCTTCCTCGCCACATCGCCACCTTCACATAGCATTTGCCATTTGGCCGAGGAACCCTGCAAACCTCCATTTCTTCATTGTGGATGGAACTCCAGTCAGAGAGTTCACGAACATGGAGTCAATTGGTGTTCCATTCCCAAAGAACCAACCAATGAGGCTATACTCGAGCATTTGGAATGCTGATGACTGGGCGACAAGAGGGGGGCTCATCAAGACAGACTGGACACAAGCTCCTTTCACCACTTCATATGTGAATTTCAAAGCCGATGCATGTATATGGTCTTCTGGAGCAGCTTCTTGCGGTTCCTTGGCCTCCAGCACCCACGCTTGGATCCCGCGGGAATTAGATACCATAAGCCGAGAAAGGCTGAAATGGGTGCAGAAGAATTTCATGATCTACAACTATTGCACGGATACGAAACGATTTCCCCAGGGCTTCCCTCCAGAATGCAAAGCCACATCATTCTGA

>SsuXTH19

ATGGCTATTATTATTATGATTGTCGCTTCTTCCCTTGTTCTGAGTCTTTTCACAGTGGCCTCTGCCGGCAACTTCTACCAGGATTTCGACATCACATGGGGCGACGGCCGAGCCAACATACTCAACAACGGCCAGCTTCTCACCCTCTCCCTCGATAAAACCTCTGGCTCTGGATTCAAATCCAAAAACCAGTACTTGTTTGGAAACATTGATATGCAGCTAAAGCTCGTGCCGGGTAACTCTGCCGGGACTGTCACTGCATACTATTTGTCTTCGGTTGGATCAACTCATGATGAAATTGACTTCGAATTTCTGGGTAATTTGAGTGGCGACCCTTATATTCTCCACACCAATGTGTTTACGCAAGGCAAAGGGAACAGAGAGCAGCAGTTTTATCTTTGTTTCTCGGTGGATGGGACTCCCATTAGACAGTTCAAGAACTTGGAATCAAATGGGATTCCATTCCCTAAATACCAACCCATGTGGATATACTCCAGCCTCTGGAACGCCGATGACTGGGCGACACGTGGAGGCCTCGTAAAGACCGATTGGAGCCAAGCTCCATTCACTGCTTCCTACAGGAACTTCACTGCCCAAGCTTGCATATCATCAGGAGGAGGTTCCTCCTCCACTTCTTGTTCCAACAACTCGTCCTGGTTAACTCAGTCACTCGACTCAACTGGCCAACAGAAGATCAAATGGGTGCAAAACAACTACATGATTTACAACTACTGCACTGATACTAAACGCTTCCCTCAAGGACTTCCCCCAGAATGCTCACTATAA

>SsuXTH20

ATGGCTTCTTTTCTAATCAACCTCTTTAGCATTGTCCTAATTGCTGTCATGAGTTGTGCAAATGGAGATTCAACCTTTTATCAACTCTACAAACCTTTGTGGGGGTTTAATCATCTCACTGTTCTTAACCAAGGACAGGAAGTGCAACTCTTACTAGACACTTCCTCCGGAGCTGGATTCAGGTCCAAGTCAGATTATTGTTTTGGGTCATTTCAAATGAAGATGAAAATATCAGAGAAAAAAAGTGGAGGAGTTGTTTCTGCTTTCTATCTGACATCATCTGCGAATAATGTTGGAAACCATGATGAGATTGATTTCGAGTTTGTTGGTACGACTGGGATATTGCAGACAAATGTGTTTGCAAATGACCTTGGCCACAGAGAGGAAAGGATTCAACTCTCGTTCGATCCTTCTGCTGCGTTTCACACTTATGAAATTGTCTGGAGCCAATACCAAGTAGTGTTCAACGTGGACTCCAAGCCCATCAGAGTGTTCAAGACTAGCGCAGGAATAAATTTTTCTTCGAAGCCCACCCATGTAGAGGGAACCATATGGAATGTAAGTTGGGCCGGAACTGTTGAGTGGTCCAAAGCACCTTTCATAGTCCACTACCAAGGTTTCAACATTAATGCCCCCCAAACGAGTCTCAACCAGCGTTGTTTTCCATTAGACTCAGATTCGGGCCCAAACTCAAACTCAAACTCGACTATAAAGCCGTGGGAACTTAGCCCTGCTCAACAAGAAGAGTATGAAGAGTATAGAAGCAAATATCTGCTTTGGAATTATTGTTCAGACAAGTCAAGATACCACCCAGAATGTGAGTCGAAATAA

>SsuXTH21

ATGGCTCTCTTTCTCTCTCTTCTTCTCATTTTCATGTTCCCTTTAACCAATGCACAGGGGCCACCTTCACCAGGCTACTACCCAAGTTCCAAAATTGGCTCCATAGGGTTCAATCAAGGCTTTAGAAATCGTTGGGGTCCTCAGCACCAAAATATAGATCAGGGTCGTTCAGTGACAATTTGGCTTGATAGAAACTCAGGAAGTGGGTACAAGTCTCTTGATGCATATCAATCCGGATACTTCGGCGCTGCTGTCAAGCTTCAACCTGGTTATACTGCAGGAGTGATTACATCATTTTATCTTTCGAACAATGAAGCCCATCCGGGAAACCATGATGAGATTGATATAGAGTTCCTGGGAACAACGCCGGATAAGCCTTATGTATTGCAGACCAACGTCTACATTAGAGGAAGTGGAGATGGAAATCTTATTGGAAGAGAGATGAAGTTTCATCTCTGGTTCGATCCCACTCAAGACTTCCACAACTATGCTATACTCTGGAACCCCAGTGAGATCATATTCTTTGTCGATGATGTCCCAATTAGGAGGTACCCAAGAAAGATTGATGCAACATTTCCACTAAGACCGATGTGGGTGTATGGATCAATATGGGATGCCTCTTCCTGGGCCACCGAGGAGGGAAAATACAAAGCTGATTATCGGTACCAACCATTTGTCGGTAGGTACAGCAACTTCAAACTTGGTGGTTGCAGAGCCAACGGCCCTGCCTCGTGCCGCCCTCCATGGGTGTCCCCAGTGGGCTCTACTGGGCTGAGCGGCCAGCAGTACGCGGCCATGGAATGGGTTCAGAGGAACTACAAGGTGTATGACTATTGCCATGATCCAAGGAGAGACCATACCTTCATCCCCGAGTGTTAG

>SsuXTH22

ATGCATCTGGCATTTCTAGGCTTCCCAATATATTATTTTGTTGACCAATTTCCCATTCGAGTTTTCAAGAACAACAAAGGCACTGGAGTTGGCTTCCCAGCACAACCAATGCAAGTAATGGCAACCATATGGAACGGAGATAGCTGGGCCACAGATGGAGGCAAAACTAAAATTATTTGGGCTCATGCACCATTTATAACTCACTTCCGGGGCTTTGCCATTGATGGATGCCCCTTAGACAGCTCTCACAGAGGATCATGCAATTCTCCCAAGTTCTGGTGGAATGGAGAGCAGTACTGGCAACTGTCTCCTGACCAAGAAAGAAGACATCAAGTCGTAAAGCATAAGTTTATGTACTATGATTACTGCAATGACAGACCCAGGCACCCAACAATTCCCCCAGAATGCCCACAGTGA

>SsuXTH23

ATGCCAAAACAAAATGTTCCAGCTTTCATGGTTTGCTCAGCTGGCACCAGCTTCAAGCCCCCTCCAGTTGGTCATCACAAGCCCCACATCACCTCTCCCTCTCTCTCCTGTCCCTACTTCACAAGAATTTTCCTCTTCCCTTTATATCTAACACTCCATCTCTCCCCTATATGTACCTCTCCACAACCCATAACCCTCTCACCACCTTCACTCATTCAAAAACTCTCTTGTTCTCTCTCATCCTCTAAAATGGGCTCTTCTTTCGAACTTTTGTTGGGTAGTCTAATAGTAATGAGCTCTTTGATGGTTGGCTCCGGTGGAAACTTCTACCAAGACTTTGACATAACATGGGGTGATCAAAGAGCCAAGGTATTCAGTGGAGGTCAGCTTCTGTCTCTGTCTCTAGACAAGACCTCTGGCTCTGGATTTGTATCAAAGAAAGAGTATTTGTTTGGAAGGATTGATATGCAGCTCAAACTTGTTGCTGGCAACTCTGCTGGCACTGTTACTGCTTACTATATGTCATCTCAAGGACCTACTCATGACGAGATTGACTTTGAGTTTTTGGGAAACCTCAGTGGAGACCCTTACATTGTTCACACTAATGTGTTCACTCAAGGGAAAGGAAACAGGGAGCAACAGTTCTACCTCTGGTTTGACCCTACCAGAAATTTTCACACCTACTCCATTGTTTGGAACCCCCAGCAAATCATTTTCTTGGTGGATGACACTCCCATAAGGTTATTCAAGAATGCTGAAAGAATTGGTGTCCCTTTCCCTAAAAACCAACCCATGAAGATCTACTCTAGCCTCTGGAATGCCGATGACTGGGCCACGCGAGGCGGGTTAGTGAAAGCTGATTGGACAAAAGCACCATTCACAGCGTACTACCGTAAGTTCAATGTCCAAACCTGTTCCGGTTCATGCACTTCAAACACTTCTTCGTCCCCAGGTGGGGCGTGGCAGAGCCAGGAACTTGATGACTACAGCCGAAGGCGTCTGAGATGGGTTCAGAAGAATTTCATGATTTACAATTACTGCACGGATTTGAAACGCTTCCCTCAAGGCCCTCCTCCTGAGTGCAAACGTTCGAGGTTCCTCTAG

>SsuXTH24

ATGGATAGTTTACGTTCTCTCTCTCGAATCATAACTCCCTTCTCTCTCCTCTTCTTATCTCTCCTCTGCGTCGCCAACTTCGCTGCAGCTTTCAACCTCTCCACAATCACCTTCGACCAAGGCTACACGCCTCTCTTCAGCGACTTCAACATCATCCGATCCGACGACGACAAGAGCGTCTCTCTCCTCCTCAACCGCTACTCCGGCTCTGGATTTATTTCCTCGGATTACTACAATTATGGTCTCTTCAGTGCAAACATCAAGTTGCCGTCGAATTACTCCGCCGGTATCGTCGTGGCATTTTATACATCAAATGGAGACGTATTTGAGAAAACCCATGATGAATTAGACTTCGAGTTCTTAGGAAACATCCATTCGAGGCCATGGAGGTTCCAGACGAACTTGTATGGGAATGGAAGCACGAACCGTGGCAGAGAAGAGAGGTACAATTTGTGGTTCGATCCAGCCAAGGAGTTTCATCGATACACCATTCTCTGGACCTCCAAAAGCATCATATTCTACGTTGATGAAGTCCCAATCAGAGAGGTTGTGCGCAGCGAAGCCATGGGAGCCGACTACCCATCAAAGCCTATGTCCCTCTACGCCACCATATGGGACGGCTCCACCTGGGCCACCAACGGCGGCAAATACAAAGTCAACTACAGGTTCCAGCCCTTCGTCTCTGAATTCAAAGACCTCGCCCTCCAAGGCTGCGCCGTCGACCCCATCCAGCAACTCCCCGCCCCCCACTGCGACAACTCCACCGCCGCCCTTGAAGCCGCCGACTTCGCCACCATCACCCCCGAGGGCCGCAAGGCCATGAGCTGGTTCCGCGAACGTTACATGTATTATTCTTACTGTTATGATACCGTCCGGTACCCTGTGCCGCCGCCGGAGTGCGTGATTGTGCCGTCGGAGCAGCACCTGTTTAAGGAAACTGGGAAGTTGAAGGACGCTTTGAAGATGAAGTTCGGCCGCCACCCGACTCGCCACCGCCACCGTGGTCGTGGGTCAAAGCGGAGGAACCAGGTCCCGGATGCTCCAGCTGCTTCCGATATGTGA

>SsuXTH25

ATGGCAAGCTCACGAGCTCTGTTCGTCGCTTTGTTCATCTCAATGATTGTGCTTTATTCAGGATCTGTAGATGCCAACTTTCCGAAAAGCATGTATTTCAACTGGGGTGCTTATCATTCTTCAATGACGAACAATGGGAATGATCTTCAGCTCGTGTTGGATCAAACTTCAGGTTCTGGCGTCCAAACAAAGAGAGAGTTTCTATTTGGAAGCATTGAAATGCTTATCAAGTTGGTACCTGGGAATTCTGCTGGAACCGTCACAGCCTATTATATGTCCTCCACTGGAAACTGGCACGACGAGATAGACTTTGAGTTCTTGGGTAACGTTTCGGGACAACCTTACATTATCCACACAAACATCTTTACACAAGGAAAAGGAAGCAGAGAACAGCAATTCCACCCCTGGTTTGACCCAACTGCTGATTTCCACAACTACACCATTCATTGGAACCCAACAGAAGTTGTGTGGTATGTCGATAGCTTGCCTATTCGAGTATTCAGAAACTACGAGAGCGAGGGAATTCCCTACCCAAACCAACAAGGGATGAGGGTGTACTCCAGCTTATGGGATGCTGATAACTGGGCAACAAGAGGTGGCCTTGTTAAGATCGACTGGAGCTGTGCACCCTTCATAGCCAGATACAACCGGTTTAGGGCAAGGGCTTGCAAGTGGAATGGGCCAGTTAGCATTACTCAATGCGCCTCCCAAACCGCTGCCAACTGGTGGACCTCACCCACGTACAGCCAATTGACCTATGCTAAGCAGGGTCAGATGAAATGGGTCAGAGATAACTACATGATTTATGACTACTGCAAAGATACTAAGCGATTCAATGGACAAATGCCACCTGAATGCTTTAAACCACAATACTAA

>SsuXTH26

ATGGATTTTTATATGGGTTTCTTTGTAATATGCTCTGTTCTTGTTTTGGCGTCTGGGTCTTCAAGAAATCTGCCAATCCTCTCATTCGATGAAGGCTATTCACAACTTTTTGGAGAGGACAATCTTATGATCCTCAGAGGTGGCAAAGCAGTTCACATTTCTCTGGATGAACGAACAGGTTCTGGATTTGTGTCTCAGGACCTTTACCTTCATGGATTCTTCAGTGCTTCTATAATACTCCCTGCAGATTACACAGCTGGTGTTGTTGTTGCATTTTATATGTCAAATGGAGACGTATATGAGAAGAACCATGATGAATTGGACTTTGAGTTATTGGGGAATATAAGAGGCAAAAACTGGAGAATTCAGACCAATGTTTATGGGAATGGAAGCACAAGCGTTGGGAGAGAAGAGAGATATGGCCTGTGGTTTGATCCTTGTGAAGATTTCCATCAGTACAGTATTCTGTGGACTGAGAATCAGATTGTATTTTATGTTGACAATGTTCCCATCAGAGAGATCAAAAGGACAGAATCAATGGGTGGGGACTTTCCATCCAAGCCAATGTCTTTGTATGCCACAATATGGGATGGTTCTGACTGGGCAACAAATGGGGGCAGATACAGAGTTAATTACAAATATGCCCCTTACATTGCCAAATTCTCCGACTTCATCCTACATGGCTGTGCAGTCGACCCAATTGAGCACTTATTTAAATGTGACAATGCCCCAAATTCTAAATCTATTCGTACCGGTATAATCACATCCCAACAAAGATCCCAAATGGAGAATTTCAGGAAGAGGCATATGCAATATTCCTATTGTTATGACCGGATTCGATACAAAGCTCCTCCACTTGAATGTATGATTGATCCCCAGCAAGCTGAGCGGCTCCAAGACTTTGATCCTGTTACATTTGGAGGAGTGCGCAGCCACCATGGTAAACGACACCACAGGAGCCGATCAAGCTGGACTGAGGCAACTGCTATTTGA

>SsuXTH27

ATGGCTGCTTCTTATCGTTCTCTTTCAATGTCTACGGTGTGTGTGATATTGGTTCTCTCTGTTGCTGTTCTATTGGTGGGTTCGGTTAGGTCATCTAGGTTTGATGAGTTGTTTCAGCCGAGTTGGGCATTCGACCATTTCACCTATGAAGGAGAGCTTCTCAAGATGAAACTGGATAACTATTCTGGTGCTGGATTTTCATCCAAAAGCAAGTATATGTTTGGGAAAGTCAACATTCAGATCAAGCTCGTGGAGGGTGACTCTGCTGGAACTGTCACTGCTTTCTATATGTCATCGGATGGTTCAAATCACAACGAGTTCGATTTCGAGTTCCTGGGCAACACTACCGGTGAGCCCTACCTGGTTCAGACCAACGTTTACGTCAATGGCGTGGGCAACAGAGAGCAAAGGCTGAACCTTTGGTTCGACCCCACGAAGGACTTCCACTCATACTCCCTCCTCTGGAACCAGCGCCAAGTTGTATTTTTGGTGGATGACACACCTATTCGGGTACACTCCAATTTGGAACACAGAGGCATACCTTTCCCGAAGGACCAACCGATGGGTGTTTACAGCTCAATTTGGAACGCCGACGATTGGGCGACTCAGGGCGGTCGTGTCAAAACCAACTGGATCCATGCACCCTTCGTAGCCTCCTATAGGGGCTTTGAAATTGATGCCTGTGAGTGCCCGGTCACAGTGGCGGCTGCCGACAATGCACGGCGGTGCAGTAGCAGCGGCCAGAAGAGGTACTGGTGGGACGAGCCGACAATGGCGGAACTGAACGTGCACCAGAGCCACCAGCTGATGTGGGTCAGGGCCAAACACTTGGTGTATGACTACTGCACTGACACTGCTAGGTTCCCAGTCATTCCGGCGGAGTGTGAGCACCACCGCCACTAG

>SsuXTH28

ATGTGTTCAAGTTCAGGTTCTTCAAAAATGGTTTTGGTTGTGATGTTGATGATGAGTTGCGTAATGGCTGCCTCAGCAACAATTTGCAACTTCTACCAAGATATTGACATCAATTCGGCTGACAATCAAAATGTTAAGATTTTCAACAGCGGCGAACTTTTGACTCTGACTCTCGACGAATTCTCTGCCTCCGGTTTTCAATCCAAGGCTGATTACTTATTCGCAAGGGTCGACATTCAACTCATGCTTGTCCCTGGCAACTCTGCCGGCACTGTCACCACTTGTTATTTATCTTCTCAAGGGCCAGCACACGATGAGATTGACTTCGAGTTCTTAGGCAATGTCTCTGGACAGCCATACACAGTCCATACCAACATCTACGCCAAAGGGAAAGGGAATCGCGAACAACAGTTCCATCTCTGGTTCGATCCCACAAAAGCCTTCCACACCTACTCAGTCATTTGGAACCCTAAACACATCATTTTCATGGTGGATAATATTCCAATAAGGGTATTCGACAACAATGAAGCGATCGGTGTTCCGTTCCCCAATAGCCAGCCAATGAAGATCTACGCGAGCCTTTGGAATGCCGATAGCTGGGCAACACAAGGCGGGAGAGTGAAGACCAATTGGACATGCGCTCCTTTCATTGCCTCGTACAGAAACTTCAATGTGGATGCTTCTTCTAACACCGACAATAAGTCTACCTCATTCACAAATCAGGCATGGCAAACTCAACAACTCGACTCCATGGGTAGGAAAAGACTGCGATGGGTGCAGCGCAACTACATGATTTACAATTACTGCACTGATTACGAAAGGTTTCCTCAAGGCCTTCCCCTCGAATGCAAGCGTTCGAAGCTCCACTAG

>SsuXTH29

ATGAAATTGTCTTCTTTCTATATTTTGGCTATGCTTTTGACATATCTACTCATTACTTCCCTAATTAAGGTTTGTTCAGGTGGAAATTTCTACCAAGACATTGATATCACTTGGGGTGACCAACGAGTTCAGATAATCGATGGAGGCCAACTTCTCACACTCTCTCTCGATAAGTTTTCTGGATCTGGTTTTCAATCCAAGAATCAGTACCTATTTGGCAGGATCGACATGCAACTCAAGCTTGTTCCTGGCAACTCTGCTGGTACCGTCACTACATACTACTTATCTTCTCAAGGAGCAACGCATGATGAGATCGATTTCGAGTTCTTGGGAAACCTGTCAGGAGATCCATACATATTCCACACCAATATTTTCGCACAAGGGAAGGGGAACAGGGAGCAACAGTTCTACCTCTGGTTCGACCCAACAATTGCCTTCCACACCTATTCCATCGTCTGGAACCCTCGACGCATCATTTTCATGGTGGATAATATTCCGCTAGGAGTGTTCGATAACAACGAAGCAATAGGCGTTCCATTCCCGAACAGCCAACCCATGAGATTATACTCGAGCCTTTGGAATGCAGACGACTGGGCAACACAAGGTGGTAGGGTGAAGACTGACTGGACTAAAGCTCCTTTTACGGCCTCCTACAATGTTTTCAATGCCAACGCTTGTGTTTGGTCATCTAATAATAATACATCATCGTGTGGGTCTAATTCCCATTCCATGACAAGCACTACTTCTACTTGGATGAACCAAGAAATTGATGCTATGGGCAAAAAGAGACTTCTATGGGTTCAAAAGAAGTACATGGTTTATAACTACTGCACTGATTTCAAACGTTTCCCTCAGGGTCTCCCTCCCGAATGCAAGCAATCCAGGCTCCACTAA

>SsuXTH30

ATGAATCGTTTACGTTGTTGTTCTCTCTCTAGAACAACGACAAATCATGTCTCTCTCCTCTTCTTCTCTCTCTTGTTTTTATGCATCGCCAACTTGGCATCAGCAGCAGCAGCATTCAACCTGTCAACCATAACCTTCAGCCAAGGCTACACCCCTCTCTTCAGCGACTTCAACATCCTCCGATCCTCCGATGACCGGACCGTCTCTCTCCTCCTCAACCGCTACTCCGGGTCTGGTTTTATTTCGTCGGATTACTACAATCATGGTCTGTTCAGTGCAAAAATCAAGTTACCATCGAATTACTCTGCCGGAATCGTCGTTGCATTTTATACATCAAATGGAGATGTATTTGAGAAAACCCATGATGAATTAGACTTCGAGTTCTTAGGAAACATCCGAGGGAAGCCATGGAAGTTCCAGACCAACTTGTACGGAAATGGAAGCACAAACAGGGGCAGAGAAGAGAGGTACACACTTTGGTTCGATCCAACCAAAGAGTTCCATCGATACACCATTCTCTGGACCTCCAAAAACATCATATTCTACATTGATGAAGTTCCGATAAGGGAGGTGGTGCGCAGCGACGCCATGGGCAGCGACTACCCATCAAAACCAATGTCCTTATACGCCACCATCTGGGACGCCTCCACCTGGGCCACCTCCGGCGGCAAATACAAAGTCAACTACGCCTACCAGCCCTTCGTCTCCGACTTCAAAGACCTCGTCCTCCAAGGCTGCGCCGTCGACCCAATCCAACAACTCCCCTCCGCCGCCTTCACCTGCGACGACAAGACCGCCGACCTCGAGGCCGCCGACTACGCCACCATCTCGCCTGAGGGCCGCAAGGCCATGAGCTGGTTCCGAGAAAGATTTATGTATTACTCGTATTGTTATGATACGGTGAGGTACCCTGTGCCGCCGCCTGAGTGCGTGATTATGCCTTCGGAACGGCACTTGTTTAAGGAGACCGGAAGGCTGAAGGCGGCGTTGAGAATGAAATTCGGCCGCCACCAGAACCACGGCCGTCGTAGCTCGAGGCGGAGGAGCCGGGTCCCGGCAGCTTCAGGCGCCACCATCATCACCATGTGA

>SsuXTH31

ATGGATTCTCATCTGGGTTTCGTTGTGATATGCTCTGTTCTTGTTTTGGCTTCTGGGTCTTCAAGAAATCTTCCAATCCTATCATTTGATGAAGGGTATTCACAGCTTTTTGGTGAGGACAATCTCATGGTCCTCAGGGATGGCAAATCCGTCCACATCTCTCTAGATGAACGAACAGGCTCTGGATTTGTGTCTCAAGACCTTTACCTTCATGGATTCTTCAGTGCTTCAATCAAACTGCCTGCAGATTACACAGCTGGTGTTGTTGTTGCATTTTATATGTCAAATGGAGACATGTATGAGAAGAACCATGATGAACTGGACTTTGAATTTTTGGGGAATATCAGAGGCAAAGATTGGAGAATTCAGACCAATGTTTATGGCAATGGAAGCACAAATGTTGGCAGAGAAGAGAGATATGGCCTATGGTTTGATCCTTCTGAAGATTTCCATCAATACAGTATTCTCTGGACTGATAATCAGATCATATTTTATGTTGACAATGTTCCCATAAGAGAGATCAAAAGGACAGAAGCAATGGGAGGGGACTTCCCATCCAAGCCAATGTCTTTGTATGCCACAATATGGGATGGTTCCAATTGGGCCACAAATGGTGGCAGATACAAAGTCAATTACAAATATGCCCCTTATATCGCCAAATTCTCGGACCTCGTCCTTCATGGTTGCGCGGTCGACCCGATCGAGCAATCATCAACCAAATGTGACCATTCCTCGAATTCCAAATCCATTCCTACTGGAATCACACCCGACCAACATTCCAAAATGGAGAATTTCAGGAAGAACTATATGCAATATTCCTATTGCTATGACCAGAATCGATACAAGGTTCCTCCCCCTGAATGTGTGATAAATTCCCAAGAAGCTCAGCGGCTTCGGGGTTTTGATCCTGTCACATTCGGAGGAGCTCGCCGCCACCATGCAAAGCGACGCCATCGAAGCAGATCAAGCAGGGGTGAGGCAATTGCTATTTGA

>SsuXTH32

ATGGCTCTCTTTCTCTCTATTCTTCTCATTTTCATGTTCCCTTCTTGCAATGCTGATGGTCCACCCTCACCTGGCTACTACCCTAGTTCCACAATCAGTTCAATGGGGTTTAACCAAGGTTTTAGAAATCTTTGGGGTCCTCAGCACCAAAATGTAGACCAGGGATCTTTAACAATTTGGCTTGATATAAGCTCAGGAAGTGGGTTCAAGTCACTGAGTCCGTATCAATCCGGATACTTTGGGGCTGCTGTCAAGCTTCAACCCGGTTATACTGCAGGAGTTATTACATCATTTTATCTTTCGAACAATGAAGATCATCCGGGAAACCACGACGAGATCGATCTCGAGTTCCTCGGAACCACGCCGGATAAGCCTTACGTGTTGCAGACAAACGTGTTTTTTAGAGGAACTGGAGATGGAAATATTATTGGAAGAGAGATGAAGTTTCACCTTTGGTTTGATCCTACACAAGATTTCCACAACTATGCCATACTCTGGAACCCTAGTGAGATCATATTCTTTGTGGATGATATCCCAATTAGGAGGTACCCTAGAAAAACTGACGAAACATTTCCGCTAAGACCCATGTGGGTCTATGGGTCCATATGGGATGCCTCGTCTTGGGCCACCGAAGGGGGCACATACAAGGCAGATTATAAATACCAACCCTTCATTAGTAGGTATAGCAACTTCAGGCTCGGTGGGTGCACCAGTGATGGACCTGGCTCGTGTCAACCTCCTTCCGGTTCACCATCCGGCTTAAGCGGTCTGAGCCAACAGCAGAATGCGGCTATGGAATGGGTCCAGAGGAACCAGAAGGTGTATGATTATTGCCAGGATCCAAAGAGAGACCATACCCTCTTACCTGAGTGTTAG

>SsuXTH33

ATGGCTTCAACAAAAACCATGATCAAATATCTGCGACTCGTGACCTTCATTGGGGTTCTTATTATGAATGTGGTTCAAATTTCAGTGGCTTCCGTCGTGTCGACTGGAGATTTCAGCAAGGATTTCTTCGTGTTATGGTCTCCTACCCACGTAAACACTTCTGCAGATGGTAGTGCAAGAAGCTTGAAGCTTGACAAAGATTCAGGCTCTGGATTTGCCTCCAACGACATGTTCTTGTTTGGTCAGATTGACATGCAAATTAAGCTGATACCTGGTGACTCGGCAGGAACAGTCTTGGCCTTTTATCTAACATCCGATCAGCCGAATCGGGATGAGATAGACTTCGAGTTCCTTGGCAATGTCAGCGGGCAGCCTTACATCATTCAAACAAATGTTTTTGCAGATGGGTTTGACAACAGAGAAGAGAGAATCTATCTCTGGTTCGATCCAACAGAGGACTTCCACACCTATTCCATCCTTTGGAACCTTCATCAAATTGTTTTCATGGTAGATTGGGTTCCCATTAGAACATACAGAAACCATGCAGACAAGGGAGTGGCATTTCCAAGGTGGCAGCCAATGAGCATAAAAATCAGCCTATGGAACGGAGACACGTGGGCAACACGTGGTGGCAAAGACAAGGTTGACTGGTCAAAGGGTCCCTTCATAGCCTCATTCAGGAACTACAAGATTGATGCATGTGTGTGGAAAGGAAATGCAAGGTTTTGTAGGGCAGATAGCCCTACAAATTGGTGGAACCAGGACATGTCAAGCTCTCTAACATGGGCACAGAGGAGGCTTTTCAAGTGGGTTAGGAAGTACCATCTCATATATGACTATTGCCAAGACAACAAGAGATTCCAAGACAACCTTCCAAAGGAGTGCTCTCTTCCCAAGTATTGA

>SsuXTH34

ATGGCCACAATGGGACACCCATACCCAACCTTGAGAAATATTGTTGTCTTTCTCTTCATACGTGTCCTCCTAGCCTTCTCGGTTTCGGGACGACCGGCCTCTTTTGTGCAGGACTTCAGAATTACGTGGTCTGATTCACATATCAGGCAAATCTATGGAGGGAGGGCCATCCAACTAGTTCTAGATCAAAACTCCGGTTGTGGGTTTGCCTCCAAGAGCCAATACTTGTTTGGACGTGTTAGCATGAAGATCAAGCTCATTGCTGGGGACTCTGCCGGGACTGTCACCGCCTTTTACATGAATTCGAACACCGACAATGTACGAGACGAGTTGGATTTTGAGTTCTTGGGGAATAGGACAGGACAGCCGTACACAGTACAAACCAATGTGTATGCACATGGAAAGGGAGATAGGGAGCAAAGGGTCAACCTTTGGTTCGATCCTGCCGCAGACTTCCACACTTACACAATCCTCTGGAACCATCATCATGTTGTGTTTTACGTGGATGCAATACCCATCAGGGTATACAAGAACAATGAAGCTAGAGGAATCCCATTCCCAAAGTTCCAACCCATGGGAATCTACTCCACACTGTGGGAAGCTGATGATTGGGCAACAAGGGGTGGACTTGAAAAGATCGATTGGACCAAAGCCCCTTTCTATGCCTACTACAAGGACTTTGACATTGAGGGTTGCCCTGTCCCGGGACCCTCTACTTGTGCTTCTAACCCATCCAATTGGTGGGAGGGTGTTGCTTACCAACAGCTTACCCCCACCCAAGCCCGAAGTTACAGGTGGGTTCGCACGAACCACTTGATCTATGACTACTGCACCGACAAATCTCGGTACCCCGTTACCCCACCGGAATGCGTGGGAGGGATCTGA

**Amino acid sequence**

>SsuXTH01

MATLAYPSFVVSLFIHVLAFSLSVSGRPATFLQDFRITWSDSHIRQIDGGRAIQLVLDQNSGCGFASKSQYLFGRVSMKIKLIPGDSAGTVTAFYMNSDTDTVRDELDFEFLGNRTGQPYTVQTNVYAHGKGNREQRVNLWFDPAADFHTYSILWNHHHVVFYVDQVPIRVYKNNEARGLPYPKLQPMGVYSTLWEADDWATRGGIEKIDWSKAPFYAYYKDFDIEGCPVPGPTTCASNSANWWEGTTYQQLSPTEARSYKWVRMNHMIYDYCVDKSRYPVTPPECVAGI*

>SsuXTH02

MCEMTNILHIENIQSQRQHKKKKRKKVEKGLPDESSQRRKEGNENDNRNSRKGPKRSTPLARIFTSNSIGVEQQLVVNNHGDGERQVRVGKGWGWVGEDCQWVEGGPELLLRSCRFSVDGTPIREFKNAESIGVPYPKNQPMRIYSSLWNADDWATRGGLVKTDWTQAPFTSSYRNFNADACIWSSGSSSCSSSSTPSSTSGTANSWLSQELDSASQDRLRWVQKNYMIYNYCNDTNRFPKGLPLECSMS*

>SsuXTH03

MASSQALLLAIFISAIAFHSRSVDANFGESMYFNWGAHHSWMGNNGKDLQLVLDQSSGSGVQTKNAYLFGSIEMLIKLVPGNSAGTVTAYYMSSTGDKHDEIDFEFLGNSSGQPYIIHTNIFTLGIGNREQQFHPWFDPTADFHNYTIHWNPTEVVWYVDSVAIRVFRNYQSEGIPYPNEQGMRVYSSLWNADNWATRGGLVKINWNSAPLIARYRNFRARACKWNGPISISQCAFQTPANWWTSPTYSQLSYAKQGQMKWVRDNYMIYDYCKDTKRFNGQMPPECFKPQY*

>SsuXTH04

MDSFPSLSKACLSFLLLLLHLLSATANAAFNVPTISFNKGFNPLFGHSNLIRSSDDKSVGLVLNRYTGSGFKSSELYNHGFFSAKIKLPSEYTAGVVVAFYTSNGDIFEKTHDELDFEFLGNIRGKRWRFQTNVYGNGSTSRGREERYTLWFDPSKEFHRYSILWTNNIIIFYIDDIPIREIVRSDAMAGDFPSKPMSLYATIWDASNWATSGGKYKVNYKYAPFVAKFTDFSLHGCASDPIQEVLSSSCSQKDDQLESTNYAHIAPKQRVAMKKFRQKYMYYSYCYDTVRYPIPPPECVIDPSMRQQFKDTGRLKFDERRRHHSKRRNQVSSMKNYGDQDED*

>SsuXTH05

MCILPSINHPLQQNSIKTKMMKYLCLLAIISVLATHLVQTSEASLVSKGDFDKDFLVIWSPSHVNTSADGLVRSLKLDNDSGSGFASNDTFLFGQFDMQIKLIEGDSAGTVVAFYLQSNQSNGDNEMDFEFLGNVIGQPYILQTNVILDGNGNREERIHLWFDPTKDFHTYSILWSLHQIVFMVDWVPIRTYRNYTDKGVSFPQWQPMRLLISLWDGDSWATRGGRDKIDWSKGPFIASFKNYKIDACVWNGDAKFCRENNSSNWWHNDRFSTLTGTQRRLFKWVRKYHLIYDYCQDNVRFQNQLPMECYLSKY*

>SsuXTH06

MGLQSRNTNPSRSSSFLPPGCVPILVFFSVFAFFFLFNLDTFLSRTKAIARHSFNEQTRLFMNAEGGNLEREFDITWGDGRAKIHNNGGLLTLSLDKNSGSGFRSKNEYLFGKIEMQIKLISGNSAGTVTTYYLSSEGPTHDEIDFEFLGNSSGDPYTLHTNVFCQGKGNREQQFFLWFDPTLDFHTYSILWNPYRIVFYVDGTPIREFKNLESKGIPYLKDQPMRIYSSLWNADDWATKGGLVKTDWKKAPFTASYRNYKPNACVWSEGTSSCSSSSLTSNSTNYSWMSEKLNSTGLERMKWVQKNHMVYNYCIDKMRFPEGLAPECKLS*

>SsuXTH07

MERGFSPMATLLCIAVLIAAYCLSVSQSQSTTPFQDNFSIMWSENHFKTSPDGQIWYLSLDNDTGCGFQTKQRYRFGWFSMKLKLVGGDSAGVVTAYYMCSENGAGPERDEVDIEFLGNKTGQPYLIQTNVYKNGTGNREMRHMLWFDPTEDFHSYSILWNTHQLVFFVDQVPIRVYKNANYTNNFFPTEKPMYLFSSIWDADNWATRGGLDKTNWTNAPFVSSYTDFSVDACQWVDPYPACVSTTTNNWWDQYAAWHLSDQQKTDFAWVERNLVVYDYCQDNKRFPTIPEECSLSPWD*

>SsuXTH08

MVICISCKASVMLLASLIVSCSFIVASASNFYDDFDITWGDQRAKIRNGGQLLTLSLDNASGSGFQSKREYLFGRIDMQLKLVSGNSAGTVTAYYLSSQGSTHDEIDFEFLGNVSGQPYIVHTNVFSQGKGNREQQFYLWFDPTRNFHTYSVVWNPQNIIFMVDNKPIRVFKNEEATTGVPYPKNQPMRIYSSLWNADDWATQGGLVKTDWSKAPFTAFYRNFKADACVPSSSSSCSSLFTTSSVSGGAAAEWQTQGLDAAGRKRLRWAQKYYMVYNYCSDLKRFPQGLPPECTR*

>SsuXTH09

MGAAPRKPVDVPFGRNYAPTWAFDHIKYFNGGSEIQLYLDKYTGTGFQSKGSYLFGHFSMQIKMVPGDSAGTVTAFYLSSQNSEHDEIDFEFLGNRTGQPYILQTNVFTGGKGDREQRIYLWFDPTKEYHSYSVLWNLYQIVFFVDDVPIRVFKNCKDLGVRFPFNQPMKIYSSLWNADDWATRGGLEKTDWSKAPFVASYKSFHVDGCEASVEAKFCDTQGKRWWDQKEFQDLDPAQYRRLQWVRQKYTIYNYCSDRTRYSTIPPECKRDRDI*

>SsuXTH10

MAFFLPLLLILMVPSTTHASYWPPSPGYYPSTKFRSMTFYQGFRNLWGPQHQRMDSNALAIWLDSTSGSGFKSVRPFRSGYFGANIKLQPGYTAGVITAFYLSNNEAHPGYHDEVDIEFLGTTFGKPYTLQTNVYIQGSGDGRIIGREMKFHLWFDPTKAFHHYAILWSPRELIFLVDDVPIRRYPKKSVATFPLRPMWLYGSIWDASSWATEEGKYKADYRYQPFVGMYNNFKATGCSAYAPRWCHPVSASPYRSGDLTRRQYMTMRWVQTHSLVYDYCKDPKRDHSLTPECWG*

>SsuXTH11

MAFSNNGFSFSMLLLVILMGMSSLMVGYGGNFYQDFDITWGGSNQRAKMFNGGQLLTLSLDKISGSGFRSKKDYLFGRIDMQLKLVSGNSAGTVTAYYLSSEGPTHDEIDFEFLGNLSGDPYILHTNVFTQGKGNREQQFYLWFDPTKNFHTYSIIWNPHNIIFMVDYTPIRVFKNGESIGVPFPKNQPMKIYSSLWNADDWATRGGLVKTDWTKAPFTAYYRNFNVQTCSGTCTSSSTSSLSNGAWQSQELDAYSRRRLRWVQKNFMIYNYCTDRRRFPQGLPPECRQRRFL*

>SsuXTH12

MAFFLSLLLILFFSSSNAAYWPPSPGYYPSSKFRSMTFYQGFRNLWGPEHQRLDNNALTIWLDRTSGSGFKSVRPFRSGYFGASIKLQPGYTAGVITAFYLSNNEAHPGYHDEVDIEFLGTTFGKPYTLQTNVYISGSGDGKIIGREMKFHLWFDPTKGFHHYAILWSPKEIIFLVDDVPIRRYPRKSVATFPLRPMWVYGSIWDASSWATEDGRYKADYRYQPFVGMYNNFKATGCSAYAPAWCRPVSASPFRSGGLSRGQHMAMRWVQTHYLVYNYCRDPKRDHSLTPECWG*

>SsuXTH13

MRIYSSIWSVDDWATRGGLVKTDWTKAPFTASYENFNANACIWSSGSSSCNSSSSKSNAWFSEQLDSTKQGREFKNAEAVGVPYPKNQPMRIYSSLWNADDWATRGGLVKTDWTKAPFTASYRNFNANACIWSSGSSSCSSSSSKSNAWLSEQLDSTNQGRLKWVQKNYMIYNYCTDSKRFPQGFPPECSMS*

>SsuXTH14

MAASSSSSSSSSSCSSVLLMISAIVFSSVLAVVSAGNFNQDFTLTWGDGRAKILNNSQLLTLSLDKTSGSGFQSNSEYLFGKIDMQLKLVPGNSAGTVTAYYLSSQGSTHDEIDFEFLGNLSGDPYILHTNVFSQGKGNREQQFYLWFDPTADFHTYSILWNPQRIIFSVDGTPIREFKNAESVGVPYPKNQPMRIYSSLWNADDWATRGGLVKTDWTQAPFTASYRNFNANACTWSSGSSSCSSSSSSTSKSNAWLSEQLDSTNQGRLKWVQKNYMIYNYCTDSKRFPQGFPPECSMS*

>SsuXTH15

MASSPSSSCSLVLLMISAIIFSSVLAIVSAGNFNQDFTITWGDGRAKILNNSQLLTLSLDKTSGSGFQSNNEYLFGKIDMQLKLVPGNSAGTVTAYYLSSQGSTHDEIDFEFLGNLSGDPYILHTNVFSQGKGNREQQFYLWFDPTADFHTYSILWNPQRIIFSVDGTPIREFKNAESVGVPYPKNQPMRIYSSLWNADDWATRGGLVKTDWTKAPFTASYRNFNANACIWSSGSSSCSSSSSKSNTWFSEQLDSTNQGRLKWVQKNYMIYNYCTDSKRFPQGFPPECSMS*

>SsuXTH16

MAASSSSSSSSSSSSSSCSSVLLMISAIVFSSVLAVVSAGNFNQDFTLTWGDGRAKILNNSQLLTLSLDKTSGSGFQSNSEYLFGKIDMQLKLVPGNSAGTVTAYYLSSQGSTHDEIDFEFLGNLSGDPYILHTNVFSQGKGNREQQFYLWFDPTADFHTYSILWNPQRIIFSVDGTPIREFKNAESVGVPYPKNQPMRIYSSLWNADDWATRGGLVKTDWTQAPFTASYRNFNANACIWSSGSSSCSSSSSSSSKSNAWLSEQLDSTNQGRLKWVQKNYMIYNYCNDSKRFPQGFPPECSMS*

>SsuXTH17

MASSSSSRVSSVLLLMMSLVVGSLFIVGSASNFNQDFDITWGNGRAKILNNGNLLTLSLDKPSGSGFQSKNEYLFGKIDMQLKLVPGNSAGTVTAYYLSSQGSTHDEIDFEFLGNLSGDPYILHTNVFSQGKGNREQQFYLWFDPTADFHTYSILWNPQRIIFSVDSTPIREFKNAESIGVPYPKNQRMRLYSSLWNADDWATRGGLVKTDWTQAPFTASYRNFNANACIWSSGSSSCGSSTPSSSSNSNAWFSEQLDSTGQERLKWVQKNYMIYNYCSDVKRFPQGFPPECNIS*

>SsuXTH18

MEKKGYEVANLERQMRKGNEKMRVYNFTPSSPHRHLHIAFAIWPRNPANLHFFIVDGTPVREFTNMESIGVPFPKNQPMRLYSSIWNADDWATRGGLIKTDWTQAPFTTSYVNFKADACIWSSGAASCGSLASSTHAWIPRELDTISRERLKWVQKNFMIYNYCTDTKRFPQGFPPECKATSF*

>SsuXTH19

MAIIIMIVASSLVLSLFTVASAGNFYQDFDITWGDGRANILNNGQLLTLSLDKTSGSGFKSKNQYLFGNIDMQLKLVPGNSAGTVTAYYLSSVGSTHDEIDFEFLGNLSGDPYILHTNVFTQGKGNREQQFYLCFSVDGTPIRQFKNLESNGIPFPKYQPMWIYSSLWNADDWATRGGLVKTDWSQAPFTASYRNFTAQACISSGGGSSSTSCSNNSSWLTQSLDSTGQQKIKWVQNNYMIYNYCTDTKRFPQGLPPECSL*

>SsuXTH20

MASFLINLFSIVLIAVMSCANGDSTFYQLYKPLWGFNHLTVLNQGQEVQLLLDTSSGAGFRSKSDYCFGSFQMKMKISEKKSGGVVSAFYLTSSANNVGNHDEIDFEFVGTTGILQTNVFANDLGHREERIQLSFDPSAAFHTYEIVWSQYQVVFNVDSKPIRVFKTSAGINFSSKPTHVEGTIWNVSWAGTVEWSKAPFIVHYQGFNINAPQTSLNQRCFPLDSDSGPNSNSNSTIKPWELSPAQQEEYEEYRSKYLLWNYCSDKSRYHPECESK*

>SsuXTH21

MALFLSLLLIFMFPLTNAQGPPSPGYYPSSKIGSIGFNQGFRNRWGPQHQNIDQGRSVTIWLDRNSGSGYKSLDAYQSGYFGAAVKLQPGYTAGVITSFYLSNNEAHPGNHDEIDIEFLGTTPDKPYVLQTNVYIRGSGDGNLIGREMKFHLWFDPTQDFHNYAILWNPSEIIFFVDDVPIRRYPRKIDATFPLRPMWVYGSIWDASSWATEEGKYKADYRYQPFVGRYSNFKLGGCRANGPASCRPPWVSPVGSTGLSGQQYAAMEWVQRNYKVYDYCHDPRRDHTFIPEC*

>SsuXTH22

MHLAFLGFPIYYFVDQFPIRVFKNNKGTGVGFPAQPMQVMATIWNGDSWATDGGKTKIIWAHAPFITHFRGFAIDGCPLDSSHRGSCNSPKFWWNGEQYWQLSPDQERRHQVVKHKFMYYDYCNDRPRHPTIPPECPQ*

>SsuXTH23

MPKQNVPAFMVCSAGTSFKPPPVGHHKPHITSPSLSCPYFTRIFLFPLYLTLHLSPICTSPQPITLSPPSLIQKLSCSLSSSKMGSSFELLLGSLIVMSSLMVGSGGNFYQDFDITWGDQRAKVFSGGQLLSLSLDKTSGSGFVSKKEYLFGRIDMQLKLVAGNSAGTVTAYYMSSQGPTHDEIDFEFLGNLSGDPYIVHTNVFTQGKGNREQQFYLWFDPTRNFHTYSIVWNPQQIIFLVDDTPIRLFKNAERIGVPFPKNQPMKIYSSLWNADDWATRGGLVKADWTKAPFTAYYRKFNVQTCSGSCTSNTSSSPGGAWQSQELDDYSRRRLRWVQKNFMIYNYCTDLKRFPQGPPPECKRSRFL*

>SsuXTH24

MDSLRSLSRIITPFSLLFLSLLCVANFAAAFNLSTITFDQGYTPLFSDFNIIRSDDDKSVSLLLNRYSGSGFISSDYYNYGLFSANIKLPSNYSAGIVVAFYTSNGDVFEKTHDELDFEFLGNIHSRPWRFQTNLYGNGSTNRGREERYNLWFDPAKEFHRYTILWTSKSIIFYVDEVPIREVVRSEAMGADYPSKPMSLYATIWDGSTWATNGGKYKVNYRFQPFVSEFKDLALQGCAVDPIQQLPAPHCDNSTAALEAADFATITPEGRKAMSWFRERYMYYSYCYDTVRYPVPPPECVIVPSEQHLFKETGKLKDALKMKFGRHPTRHRHRGRGSKRRNQVPDAPAASDM*

>SsuXTH25

MASSRALFVALFISMIVLYSGSVDANFPKSMYFNWGAYHSSMTNNGNDLQLVLDQTSGSGVQTKREFLFGSIEMLIKLVPGNSAGTVTAYYMSSTGNWHDEIDFEFLGNVSGQPYIIHTNIFTQGKGSREQQFHPWFDPTADFHNYTIHWNPTEVVWYVDSLPIRVFRNYESEGIPYPNQQGMRVYSSLWDADNWATRGGLVKIDWSCAPFIARYNRFRARACKWNGPVSITQCASQTAANWWTSPTYSQLTYAKQGQMKWVRDNYMIYDYCKDTKRFNGQMPPECFKPQY*

>SsuXTH26

MDFYMGFFVICSVLVLASGSSRNLPILSFDEGYSQLFGEDNLMILRGGKAVHISLDERTGSGFVSQDLYLHGFFSASIILPADYTAGVVVAFYMSNGDVYEKNHDELDFELLGNIRGKNWRIQTNVYGNGSTSVGREERYGLWFDPCEDFHQYSILWTENQIVFYVDNVPIREIKRTESMGGDFPSKPMSLYATIWDGSDWATNGGRYRVNYKYAPYIAKFSDFILHGCAVDPIEHLFKCDNAPNSKSIRTGIITSQQRSQMENFRKRHMQYSYCYDRIRYKAPPLECMIDPQQAERLQDFDPVTFGGVRSHHGKRHHRSRSSWTEATAI*

>SsuXTH27

MAASYRSLSMSTVCVILVLSVAVLLVGSVRSSRFDELFQPSWAFDHFTYEGELLKMKLDNYSGAGFSSKSKYMFGKVNIQIKLVEGDSAGTVTAFYMSSDGSNHNEFDFEFLGNTTGEPYLVQTNVYVNGVGNREQRLNLWFDPTKDFHSYSLLWNQRQVVFLVDDTPIRVHSNLEHRGIPFPKDQPMGVYSSIWNADDWATQGGRVKTNWIHAPFVASYRGFEIDACECPVTVAAADNARRCSSSGQKRYWWDEPTMAELNVHQSHQLMWVRAKHLVYDYCTDTARFPVIPAECEHHRH*

>SsuXTH28

MCSSSGSSKMVLVVMLMMSCVMAASATICNFYQDIDINSADNQNVKIFNSGELLTLTLDEFSASGFQSKADYLFARVDIQLMLVPGNSAGTVTTCYLSSQGPAHDEIDFEFLGNVSGQPYTVHTNIYAKGKGNREQQFHLWFDPTKAFHTYSVIWNPKHIIFMVDNIPIRVFDNNEAIGVPFPNSQPMKIYASLWNADSWATQGGRVKTNWTCAPFIASYRNFNVDASSNTDNKSTSFTNQAWQTQQLDSMGRKRLRWVQRNYMIYNYCTDYERFPQGLPLECKRSKLH*

>SsuXTH29

MKLSSFYILAMLLTYLLITSLIKVCSGGNFYQDIDITWGDQRVQIIDGGQLLTLSLDKFSGSGFQSKNQYLFGRIDMQLKLVPGNSAGTVTTYYLSSQGATHDEIDFEFLGNLSGDPYIFHTNIFAQGKGNREQQFYLWFDPTIAFHTYSIVWNPRRIIFMVDNIPLGVFDNNEAIGVPFPNSQPMRLYSSLWNADDWATQGGRVKTDWTKAPFTASYNVFNANACVWSSNNNTSSCGSNSHSMTSTTSTWMNQEIDAMGKKRLLWVQKKYMVYNYCTDFKRFPQGLPPECKQSRLH*

>SsuXTH30

MNRLRCCSLSRTTTNHVSLLFFSLLFLCIANLASAAAAFNLSTITFSQGYTPLFSDFNILRSSDDRTVSLLLNRYSGSGFISSDYYNHGLFSAKIKLPSNYSAGIVVAFYTSNGDVFEKTHDELDFEFLGNIRGKPWKFQTNLYGNGSTNRGREERYTLWFDPTKEFHRYTILWTSKNIIFYIDEVPIREVVRSDAMGSDYPSKPMSLYATIWDASTWATSGGKYKVNYAYQPFVSDFKDLVLQGCAVDPIQQLPSAAFTCDDKTADLEAADYATISPEGRKAMSWFRERFMYYSYCYDTVRYPVPPPECVIMPSERHLFKETGRLKAALRMKFGRHQNHGRRSSRRRSRVPAASGATIITM*

>SsuXTH31

MDSHLGFVVICSVLVLASGSSRNLPILSFDEGYSQLFGEDNLMVLRDGKSVHISLDERTGSGFVSQDLYLHGFFSASIKLPADYTAGVVVAFYMSNGDMYEKNHDELDFEFLGNIRGKDWRIQTNVYGNGSTNVGREERYGLWFDPSEDFHQYSILWTDNQIIFYVDNVPIREIKRTEAMGGDFPSKPMSLYATIWDGSNWATNGGRYKVNYKYAPYIAKFSDLVLHGCAVDPIEQSSTKCDHSSNSKSIPTGITPDQHSKMENFRKNYMQYSYCYDQNRYKVPPPECVINSQEAQRLRGFDPVTFGGARRHHAKRRHRSRSSRGEAIAI*

>SsuXTH32

MALFLSILLIFMFPSCNADGPPSPGYYPSSTISSMGFNQGFRNLWGPQHQNVDQGSLTIWLDISSGSGFKSLSPYQSGYFGAAVKLQPGYTAGVITSFYLSNNEDHPGNHDEIDLEFLGTTPDKPYVLQTNVFFRGTGDGNIIGREMKFHLWFDPTQDFHNYAILWNPSEIIFFVDDIPIRRYPRKTDETFPLRPMWVYGSIWDASSWATEGGTYKADYKYQPFISRYSNFRLGGCTSDGPGSCQPPSGSPSGLSGLSQQQNAAMEWVQRNQKVYDYCQDPKRDHTLLPEC*

>SsuXTH33

MASTKTMIKYLRLVTFIGVLIMNVVQISVASVVSTGDFSKDFFVLWSPTHVNTSADGSARSLKLDKDSGSGFASNDMFLFGQIDMQIKLIPGDSAGTVLAFYLTSDQPNRDEIDFEFLGNVSGQPYIIQTNVFADGFDNREERIYLWFDPTEDFHTYSILWNLHQIVFMVDWVPIRTYRNHADKGVAFPRWQPMSIKISLWNGDTWATRGGKDKVDWSKGPFIASFRNYKIDACVWKGNARFCRADSPTNWWNQDMSSSLTWAQRRLFKWVRKYHLIYDYCQDNKRFQDNLPKECSLPKY*

>SsuXTH34

MATMGHPYPTLRNIVVFLFIRVLLAFSVSGRPASFVQDFRITWSDSHIRQIYGGRAIQLVLDQNSGCGFASKSQYLFGRVSMKIKLIAGDSAGTVTAFYMNSNTDNVRDELDFEFLGNRTGQPYTVQTNVYAHGKGDREQRVNLWFDPAADFHTYTILWNHHHVVFYVDAIPIRVYKNNEARGIPFPKFQPMGIYSTLWEADDWATRGGLEKIDWTKAPFYAYYKDFDIEGCPVPGPSTCASNPSNWWEGVAYQQLTPTQARSYRWVRTNHLIYDYCTDKSRYPVTPPECVGGI*
