# Supplementary material for: The XTH Gene Family in Schima superba: Genome-Wide Identification, Expression Profiles, and Functional Interaction Network Analysis
Source: Front Plant Sci. 2022 Jun 16;13:911761. doi: 10.3389/fpls.2022.911761 (PMC9243642; doi:10.3389/fpls.2022.911761)
Supplement: Supplementary file 1 [file Data_Sheet_1.ZIP › Supplementary/Supplementary Table 9.docx]

Supplementary Table 9 Predicted 3D structure of the SsuXTH proteins

| Template | Gene name | Seq Identity (%) | GMQE | QMEANDisCo | Aligment Coverage (%) | 3D Image |
| --- | --- | --- | --- | --- | --- | --- |
| 1umz.1.A | SsuXTH01 | 54.14 | 0.84 | 0.85±0.05 | 92 | 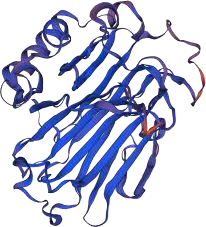 |
| 1un1.2.A | SsuXTH02 | 42.39 | 0.56 | 0.62± 0.06 | 74 | 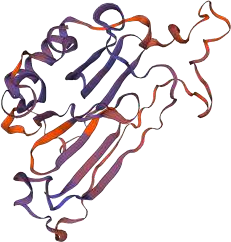 |
| 1umz.1.A | SsuXTH03 | 48.51 | 0.80 | 0.80 ± 0.05 | 92 | 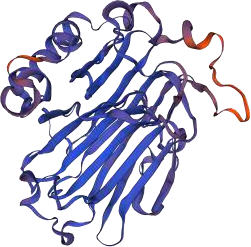 |

| Template | Gene name | Seq Identity (%) | GMQE | QMEANDisCo | Aligment Coverage (%) | 3D Image |
| --- | --- | --- | --- | --- | --- | --- |
| 1umz.1.A | SsuXTH04 | 38.02 | 0.62 | 0.73 ± 0.05 | 77 | 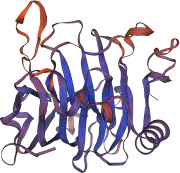 |
| 1umz.1.A | SsuXTH05 | 49.25 | 0.73 | 0.80 ± 0.05 | 85 | 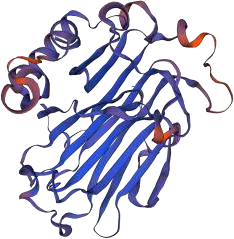 |
| 1umz.1.A | SsuXTH06 | 51.53 | 0.69 | 0.81 ± 0.05 | 79 | 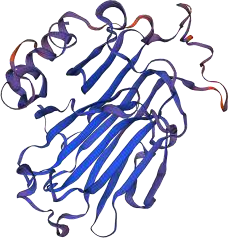 |

| Template | Gene name | Seq Identity (%) | GMQE | QMEANDisCo | Aligment Coverage (%) | 3D Image |
| --- | --- | --- | --- | --- | --- | --- |
| 1umz.1.A | SsuXTH07 | 47.21 | 0.78 | 0.80 ± 0.05 | 90 | 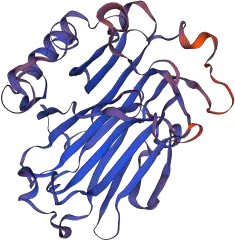 |
| 1umz.1.A | SsuXTH08 | 58.02 | 0.78 | 0.82 ± 0.05 | 89 | 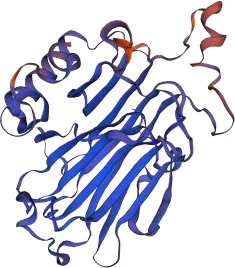 |
| 1umz.1.A | SsuXTH09 | 90.44 | 0.96 | 0.94 ± 0.05 | 99 | 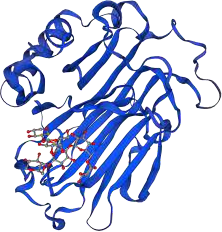 |

| Template | Gene name | Seq Identity (%) | GMQE | QMEANDisCo | Aligment Coverage (%) | 3D Image |
| --- | --- | --- | --- | --- | --- | --- |
| 2uwa.1.A | SsuXTH10 | 69.49 | 0.87 | 0.87 ± 0.05 | 92 | 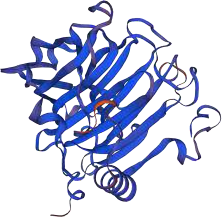 |
| 1umz.1.A | SsuXTH11 | 57.84 | 0.82 | 0.84 ± 0.05 | 91 | 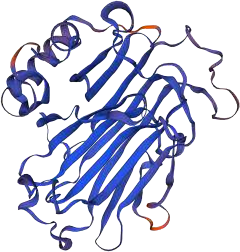 |
| 2uwa.1.A | SsuXTH12 | 70.96 | 0.87 | 0.85 ± 0.05 | 93 | 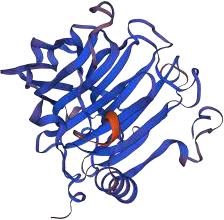 |

| Template | Gene name | Seq Identity (%) | GMQE | QMEANDisCo | Aligment Coverage (%) | 3D Image |
| --- | --- | --- | --- | --- | --- | --- |
| 1un1.2.A | SsuXTH13 | 53.72 | 0.41 | 0.52 ± 0.07 | 63 | 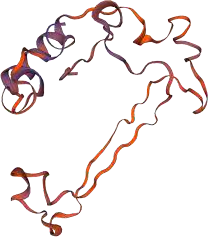 |
| 1umz.1.A | SsuXTH14 | 56.65 | 0.78 | 0.83 ± 0.05 | 88 | 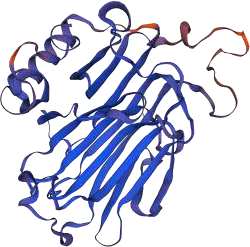 |
| 1umz.1.A | SsuXTH15 | 56.87 | 0.80 | 0.84 ± 0.05 | 90 | 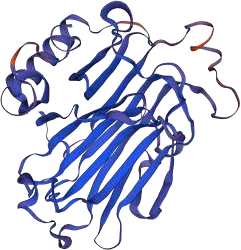 |

| Template | Gene name | Seq Identity (%) | GMQE | QMEANDisCo | Aligment Coverage (%) | 3D Image |
| --- | --- | --- | --- | --- | --- | --- |
| 1umz.1.A | SsuXTH16 | 56.27 | 0.77 | 0.83 ± 0.05 | 87 | 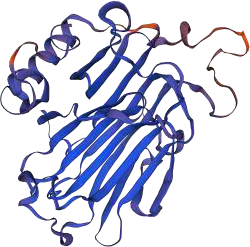 |
| 1umz.1.A | SsuXTH17 | 56.27 | 0.79 | 0.83 ± 0.05 | 89 | 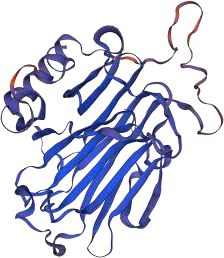 |
| 1un1.2.A | SsuXTH18 | 46.88 | 0.66 | 0.61 ± 0.07 | 87 | 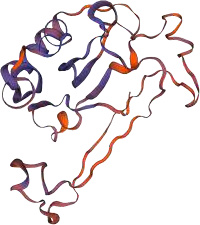 |

| Template | Gene name | Seq Identity (%) | GMQE | QMEANDisCo | Aligment Coverage (%) | 3D Image |
| --- | --- | --- | --- | --- | --- | --- |
| 1umz.1.A | SsuXTH19 | 54.96 | 0.81 | 0.80 ± 0.05 | 93 | 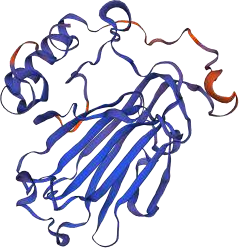 |
| 1umz.1.A | SsuXTH20 | 45.90 | 0.72 | 0.73 ± 0.05 | 88 | 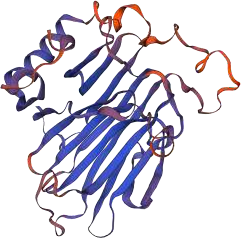 |
| 2uwa.1.A | SsuXTH21 | 75.09 | 0.89 | 0.87 ± 0.05 | 93 | 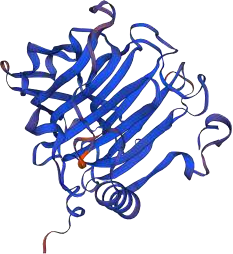 |

| Template | Gene name | Seq Identity (%) | GMQE | QMEANDisCo | Aligment Coverage (%) | 3D Image |
| --- | --- | --- | --- | --- | --- | --- |
| 1un1.2.A | SsuXTH22 | 43.28 | 0.72 | 0.66 ± 0.07 | 97 | 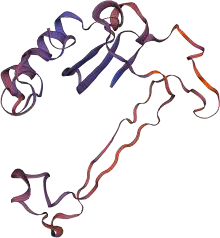 |
| 1umz.1.A | SsuXTH23 | 57.09 | 0.62 | 0.83 ± 0.05 | 73 | 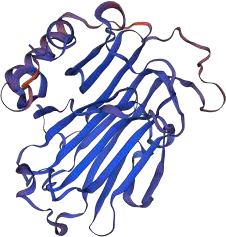 |
| 2vh9.1.A | SsuXTH24 | 40.86 | 0.61 | 0.72 ± 0.05 | 79 | 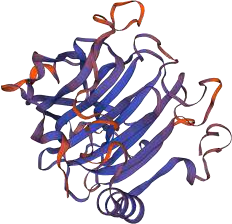 |

| Template | Gene name | Seq Identity (%) | GMQE | QMEANDisCo | Aligment Coverage (%) | 3D Image |
| --- | --- | --- | --- | --- | --- | --- |
| 1umz.1.A | SsuXTH25 | 48.88 | 0.80 | 0.81 ± 0.05 | 92 | 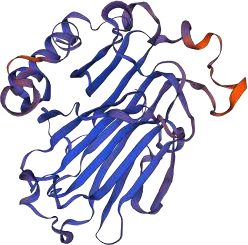 |
| 2uwa.1.A | SsuXTH26 | 43.41 | 0.64 | 0.74 ± 0.05 | 78 | 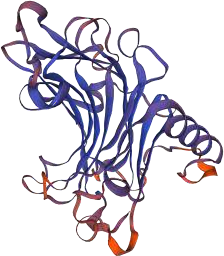 |
| 1umz.1.A | SsuXTH27 | 54.10 | 0.80 | 0.83 ± 0.05 | 89 | 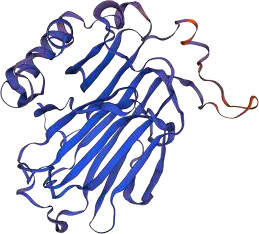 |

| Template | Gene name | Seq Identity (%) | GMQE | QMEANDisCo | Aligment Coverage (%) | 3D Image |
| --- | --- | --- | --- | --- | --- | --- |
| 1umz.1.A | SsuXTH28 | 52.29 | 0.81 | 0.83 ± 0.05 | 91 | 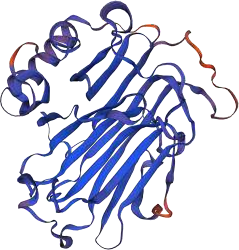 |
| 1umz.1.A | SsuXTH29 | 53.38 | 0.79 | 0.83 ± 0.05 | 90 | 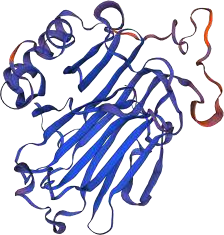 |
| 1umz.1.A | SsuXTH30 | 36.50 | 0.57 | 0.71 ± 0.05 | 73 | 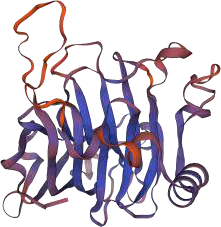 |

| Template | Gene name | Seq Identity (%) | GMQE | QMEANDisCo | Aligment Coverage (%) | 3D Image |
| --- | --- | --- | --- | --- | --- | --- |
| 2uwa.1.A | SsuXTH31 | 46.30 | 0.66 | 0.76 ± 0.05 | 78 | 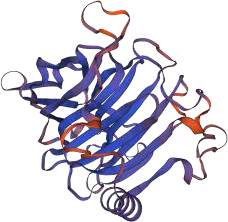 |
| 2uwa.1.A | SsuXTH32 | 75.19 | 0.89 | 0.89 ± 0.05 | 93 | 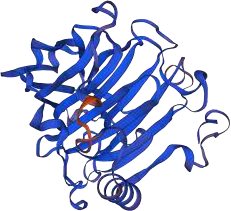 |
| 1umz.1.A | SsuXTH33 | 50.75 | 0.78 | 0.81 ± 0.05 | 89 | 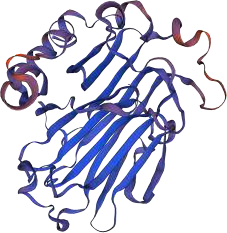 |

| Template | Gene name | Seq Identity (%) | GMQE | QMEANDisCo | Aligment Coverage (%) | 3D Image |
| --- | --- | --- | --- | --- | --- | --- |
| 1umz.1.A | SsuXTH34 | 54.31 | 0.82 | 0.84 ± 0.05 | 91 | 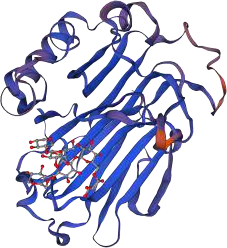 |
| The identity between the template and sequence should be > 30%; The GMQE value is [0-1], and the greater the value, the better the quality; QMEANDisCo value is in [0-1], the  larger the value, the better the matching degree. | | | | | | |
